# Identification of protein features encoded by alternative exons using Exon Ontology

**DOI:** 10.1101/gr.212696.116

**Published:** 2017-06

**Authors:** Léon-Charles Tranchevent, Fabien Aubé, Louis Dulaurier, Clara Benoit-Pilven, Amandine Rey, Arnaud Poret, Emilie Chautard, Hussein Mortada, François-Olivier Desmet, Fatima Zahra Chakrama, Maira Alejandra Moreno-Garcia, Evelyne Goillot, Stéphane Janczarski, Franck Mortreux, Cyril F. Bourgeois, Didier Auboeuf

**Affiliations:** 1 Université Lyon 1, ENS de Lyon, CNRS UMR 5239, INSERM U1210, Laboratory of Biology and Modelling of the Cell, F-69007, Lyon, France;; 2 Laboratoire de Biométrie et Biologie Évolutive, Université Lyon 1, UMR CNRS 5558, INRIA Erable, Villeurbanne, F-69622, France;; 3 Institut NeuroMyoGène, CNRS UMR 5310, INSERM U1217, Université Lyon 1, Lyon, F-69007 France

## Abstract

Transcriptomic genome-wide analyses demonstrate massive variation of alternative splicing in many physiological and pathological situations. One major challenge is now to establish the biological contribution of alternative splicing variation in physiological- or pathological-associated cellular phenotypes. Toward this end, we developed a computational approach, named “Exon Ontology,” based on terms corresponding to well-characterized protein features organized in an ontology tree. Exon Ontology is conceptually similar to Gene Ontology-based approaches but focuses on exon-encoded protein features instead of gene level functional annotations. Exon Ontology describes the protein features encoded by a selected list of exons and looks for potential Exon Ontology term enrichment. By applying this strategy to exons that are differentially spliced between epithelial and mesenchymal cells and after extensive experimental validation, we demonstrate that Exon Ontology provides support to discover specific protein features regulated by alternative splicing. We also show that Exon Ontology helps to unravel biological processes that depend on suites of coregulated alternative exons, as we uncovered a role of epithelial cell-enriched splicing factors in the AKT signaling pathway and of mesenchymal cell-enriched splicing factors in driving splicing events impacting on autophagy. Freely available on the web, Exon Ontology is the first computational resource that allows getting a quick insight into the protein features encoded by alternative exons and investigating whether coregulated exons contain the same biological information.

Alternative splicing is a major step in the gene expression process leading to the production of different transcripts with different exon content (or alternative splicing variants) from one single gene. This mechanism is the rule, as 95% of human genes produce at least two splicing variants (Nilsen and Graveley 2010; de Klerk and ‘t Hoen 2015; Lee and Rio 2015). Alternative splicing decisions rely on splicing factors binding on pre-mRNA molecules more or less close to splicing sites and regulating their recognition by the spliceosome (Lee and Rio 2015). Other mechanisms, including usage of alternative promoters and alternative polyadenylation sites, also increase the diversity of transcripts and drive both quantitative and qualitative effects (Tian and Manley 2013; de Klerk and ‘t Hoen 2015). Indeed, alternative promoters and alternative polyadenylation sites can impact mRNA 5′- and 3′- untranslated regions, which can have consequences on transcript stability or translation (Tian and Manley 2013; de Klerk and ‘t Hoen 2015). In addition, alternative splicing can lead to the biogenesis of nonproductive mRNAs degraded by the nonsense-mediated mRNA decay pathway (Hamid and Makeyev 2014). These mechanisms can also change the gene coding sequence. Alternative promoters and alternative polyadenylation sites can change protein N- and C-terminal domains, respectively, and alternative splicing can impact any protein feature (Kelemen et al. 2013; Light and Elofsson 2013; Tian and Manley 2013; de Klerk and ‘t Hoen 2015). Therefore, all these mechanisms increase the diversity of the proteome coded by a limited number of genes.

The nature (i.e., exon content) of gene products is tightly regulated, leading different cell types to express specific sets of protein isoforms contributing to specific cellular functions. For example, the selective expression of protein isoforms plays a major role in the biological functions of epithelial and mesenchymal cells, which are two major cell types found in many tissues (Bebee et al. 2014; Mallinjoud et al. 2014; Yang et al. 2016b). Epithelial and mesenchymal cells ensure different physiological functions (epithelial cells are interconnected and nonmotile cells, while mesenchymal cells are isolated and motile cells), and the epithelial-to-mesenchymal transition has been shown to contribute to metastasis formation during tumor progression (Bebee et al. 2014; Yang et al. 2016b). Several splicing factors, including ESRP1, ESRP2, RBM47, and RBFOX2, control the exon inclusion rate in an epithelial cell- or mesenchymal cell-specific manner, leading to the production of protein isoforms driving biological processes like cell polarity, adhesion, or motility (Venables et al. 2013; Bebee et al. 2014; Mallinjoud et al. 2014; Vanharanta et al. 2014; Yang et al. 2016b).

Alternative splicing plays a major role in several pathological situations, as massive splicing variation is observed in many diseases (Cieply and Carstens 2015; Daguenet et al. 2015; Sebestyen et al. 2016). However, the analysis of the cellular functions driven by specific splicing-derived protein isoforms is a major challenge for two main reasons. First, multiple splicing variants from any gene are often observed to be differentially expressed when comparing two biological situations. This creates, therefore, a problem of resource prioritization for the massive task of splicing isoform functional characterization. In this context, the selection of specific splicing variants for further functional analyses is often biased and based on the gene functions described in the literature, which puts the focus on well-characterized genes while overlooking the poorly characterized ones. In addition, the protein features affected by alternative splicing are currently mostly analyzed manually in a time-consuming process. The second challenge relies on the identification of processes impacted by coregulated exons. Indeed, the functional output resulting from splicing variant misregulation is currently analyzed on a gene-by-gene basis without considering the global impact of coregulated splicing variants. It is expected that identifying common protein features affected by splicing variations will allow a better understanding of the contribution of alternative splicing in cellular phenotypes.

In order to address these concerns, we developed and made available on the web a computational approach named “Exon Ontology,” that is conceptually similar to the Gene Ontology approach but focuses on exon-encoded protein features instead of gene-level functional annotations. This strategy allowed us to characterize individual and coregulated protein features impacted by alternative splicing of exons that are differentially spliced between epithelial and mesenchymal cells.

## Results

### Exon Ontology tree and exon annotation

**Figure 1.**
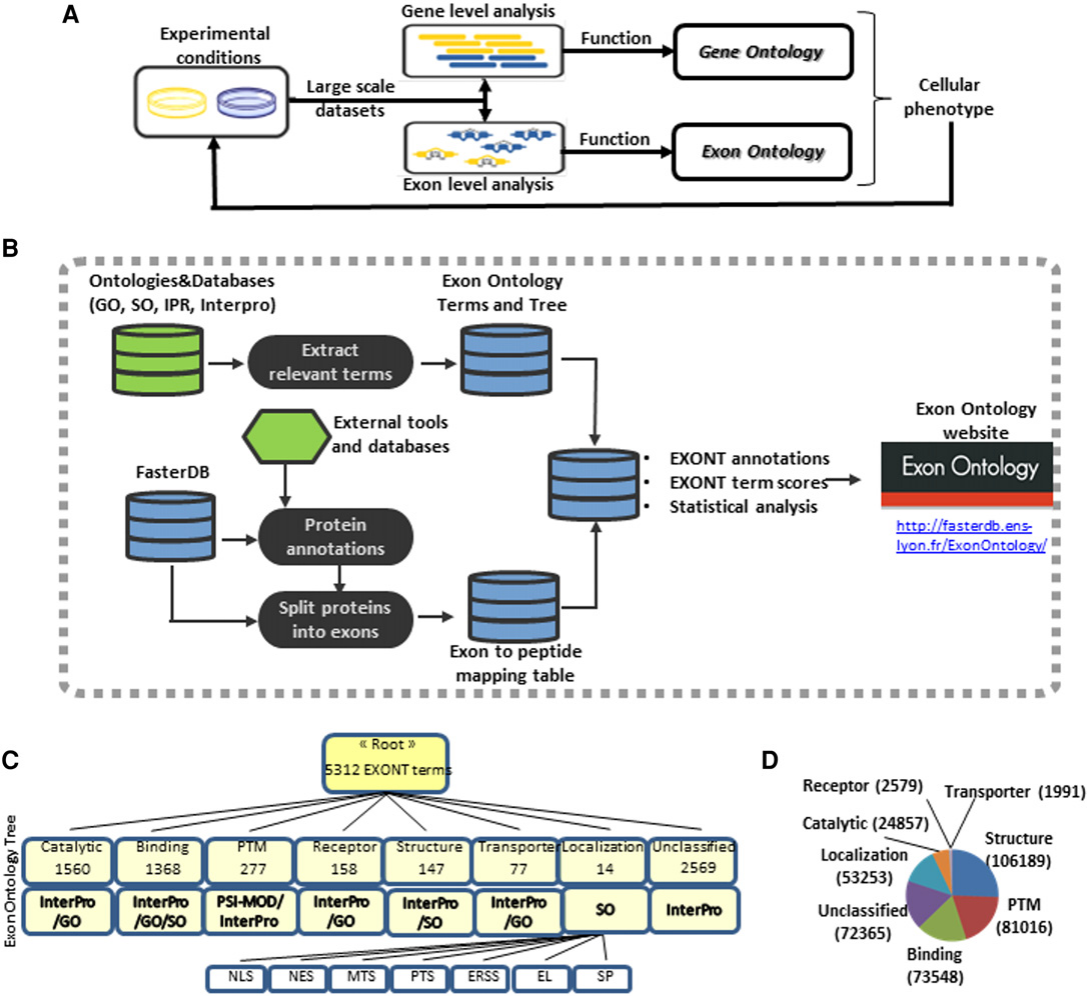
(*A*) Genome-wide transcriptomic analyses allow identification of genes whose expression levels are modified when comparing two experimental conditions. Looking for the enrichment of Gene Ontology (GO) terms associated with these genes allows prediction of the biological processes and cellular activities that are likely to be impacted by gene expression level modifications. Exon Ontology aims at identifying protein features associated with changes of exon content owing to alternative splicing regulation, which may contribute to cellular phenotypes. Both GO and Exon Ontology predictions can next be addressed by dedicated experimental approaches. (*B*) The Exon Ontology workflow is based on ontological terms (EXONT terms) that were derived from existing ontologies and databases (e.g., GO, Sequence Ontology, PSI-MOD, and InterPro). Protein features were derived from reference tools and databases and were mapped to annotated genomic exons in the ‘Faster DB’ database. Genomic exons can thus be associated with one or several EXONT terms. A computational suite (Exon Ontology) then calculates a dedicated EXONT term score and looks for potential EXONT term enrichment by statistical analysis. (*C*) Protein features and domains have been assigned Exon Ontology (EXONT) terms based on existing ontologies and databases as described in panel *B*. The EXONT terms were organized in an Exon Ontology tree based on height classes of protein features (e.g., catalytic, binding). Each class was divided in categories and contains a more or less large number of associated terms. For example, the “Localization” class was divided into “Nuclear Localization Signal” (NLS), “Nuclear Export Signal” (NES), “Mitochondrial Targeting Signal” (MTS), “Peroxisomal Targeting Signal” (PTS), “Endoplasmic Reticulum Signal Sequence” (ERSS), “Endosomal Localization Signal” (EL), and “Signal Peptide” (SP) categories based on the “Sequence Ontology” resource. (*D*) Pie chart showing the distribution of functional annotations of human coding exons; more than 170,000 human coding exons are associated with at least one Exon Ontology term. The numbers represent the number of exons associated with each of the main classes of the Exon Ontology terms.

Large-scale RNA sequencing technologies allow characterization of the expression level of cellular transcripts as well as their exon content. Computational analyses based on Gene Ontology (GO), which relies on gene functional annotations (or GO terms), allow prediction of the biological processes (enriched GO terms) that are likely to be impacted by changes in gene expression level (Fig. 1A). We developed Exon Ontology to identify protein domains and features that are impacted by alternative splicing variations (Fig. 1A). For this purpose, we defined Exon Ontology (EXONT) terms from existing databases including Sequence Ontology, Protein Modification Ontology, InterPro, and Gene Ontology (Montecchi-Palazzi et al. 2008; Mungall et al. 2011; Gene Ontology Consortium 2015; Mitchell et al. 2015). The EXONT terms were organized in an ontology tree based on eight major protein features that can be affected by alternative splicing (Fig. 1B,C). These include protein domains with catalytic, binding, receptor, and transporter activities and protein regions containing subcellular localization signals, structural features, and experimentally validated post-translational modifications (PTMs). Each class of protein features was next divided into categories based on existing ontological trees. For example, the “Localization” class was divided into eight categories using the ontology tree defined by the “Sequence Ontology” resource (SO) (Fig. 1C; Mungall et al. 2011). Categories corresponding to the “catalytic” class were extracted from InterPro and Gene Ontology (Gene Ontology Consortium 2015; Mitchell et al. 2015). A total of 5312 Exon Ontology terms was used to generate the Exon Ontology tree (Fig. 1C; Supplemental Table S2).

Meanwhile, protein annotations retrieved from reference tools and databases were mapped to the genomic exons defined in the FasterDB genome annotation database that we previously developed (Fig. 1B; Mallinjoud et al. 2014). In so doing, FasterDB genomic exons were associated with one or several EXONT terms and a web interface was developed in order to easily retrieve the EXONT terms associated with genomic exons (Fig. 1B). A large proportion of the 190,617 coding exons defined in FasterDB was associated with “Structure”-, “PTM” (post-translational modification)-, “Binding”-, “Localization”-, and/or “Catalytic”-associated terms (Fig. 1D). It is important to emphasize that Exon Ontology is based on exon-level annotations and relies neither on full-length transcript annotations nor on transcript/gene-associated GO terms. Exon Ontology allows the association of each human coding exon to the characteristic(s) or protein feature(s) it encodes for, but it does not allow one to precisely predict the impact of alternative splicing on protein cellular functions.

### Enrichment of Exon Ontology terms

**Figure 2.**
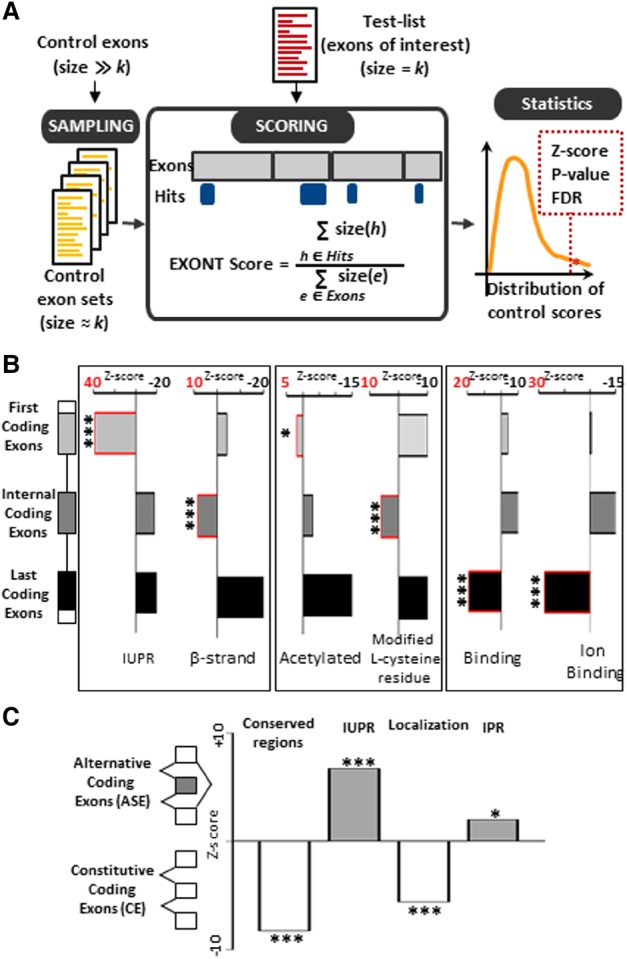
(*A*) Looking for enrichment of protein features (EXONT terms) encoded by a set of exons requires a quantitative measurement. The ‘EXONT score’ for each EXONT term associated with exons from a test-list is calculated by dividing the number of nucleotides covered by each EXONT term (size [h]) by the total number of nucleotides of each tested exon (size [e]). The enrichment *Z*-score and statistical significance are calculated by comparing the calculated EXONT score as described above to the scores obtained from a large number of sets of control exons having approximately the same size as the test-list. This analysis is available on the Exon Ontology website for the 91 EXONT terms that are most frequently associated with exons (listed in Supplemental Table S2). (*B*) Several Exon Ontology terms were enriched at either end of their mRNAs (first, internal, and last coding exons). The *x*-axis corresponds to the *Z*-scores obtained by comparing one category of exons to all exons. Positive *Z*-score values (red numbers and boxes) indicate EXONT term enrichment in the corresponding exon category. IUPR: Intrinsically Unstructured Polypeptide Region, Acetylated: Acetylated residues. (*) FDR adjusted *P*-value < 0.05, (***) FDR adjusted *P*-value < 0.005. (*C*) Constitutive and alternative coding exons are enriched for different EXONT terms. The *y*-axis corresponds to the *Z*-scores obtained by comparing alternative to constitutive exons. Positive *Z*-scores indicate enrichment of the corresponding EXONT term in alternative exons, while negative values indicate enrichment in constitutive exons. IUPR: Intrinsically unstructured polypeptide region, IPR: Intramembrane Protein Region. (*) FDR adjusted *P*-value < 0.05, (***) FDR adjusted *P*-value < 0.005.

In order to look for potential enrichment of specific protein features (or EXONT terms) within a list of coregulated exons, we established an EXONT score by measuring the coverage of each EXONT term in a list of exons. The EXONT score is defined as the number of nucleotides covered by hits of the EXONT term divided by the total number of nucleotides of all the exons from the tested list (Fig. 2A; Methods). We also established a *Z*-score associated with a statistical test by comparing the score of a selected exon set to scores of randomly built exon sets of approximately the same total size (Fig. 2A; Methods).

In an attempt to decide what kind of control exons should be used, we generated three categories of exons (first, internal, and last coding exons), as we anticipated that the protein features encoded by exons may depend on their position within the gene. We therefore calculated the *Z*-scores for each of the three exon categories by comparing its exons to all coding exons defined in FasterDB. This revealed that different EXONT terms are enriched (positive *Z*-score values) in different parts of the mRNAs, as illustrated in Figure 2B (Supplemental Table S3). In addition, when comparing annotated alternative internal coding exons (or alternatively spliced exons [ASEs]) to constitutive internal coding exons (CEs), we observed differential EXONT term enrichment and confirmed several previous findings (Fig. 2C; Supplemental Table S3). For example, there was a strong enrichment for the “Intrinsically Unstructured Protein Regions” (IUPRs) term in ASEs when compared to CEs (a positive or negative *Z*-score value means that an EXONT term is enriched in ASEs or CEs, respectively) (Fig. 2C), as previously reported (Romero et al. 2006; Buljan et al. 2012, 2013; Ellis et al. 2012; Weatheritt et al. 2012; Colak et al. 2013). Meanwhile, CEs are enriched for the “Polypeptide Conserved Regions” term when compared to ASEs, supporting previous reports indicating that CEs are often more conserved than ASEs (Plass and Eyras 2006; Lev-Maor et al. 2007; Mudge et al. 2011). Several terms from the “Localization” class were enriched in CEs when compared to ASEs. There was, however, an enrichment for several terms associated with “membrane” in ASEs (Fig. 2C, “Intramembrane Polypeptide Region” [IPR]; Supplemental Table S3). This observation suggests that alternative splicing may impact the ability of proteins to be incorporated into cellular membranes as was reported in a few cases (Stamm et al. 2005; Jones et al. 2009; Tejedor et al. 2015). Even though we do not know yet the biological meaning of the enrichment for some protein features in different exon categories, we believe these data are important to underscore the importance of using an appropriate set of control exons.

### Exon Ontology reveals specific protein features affected in exons that are differentially spliced between epithelial and mesenchymal cells

**Figure 3.**
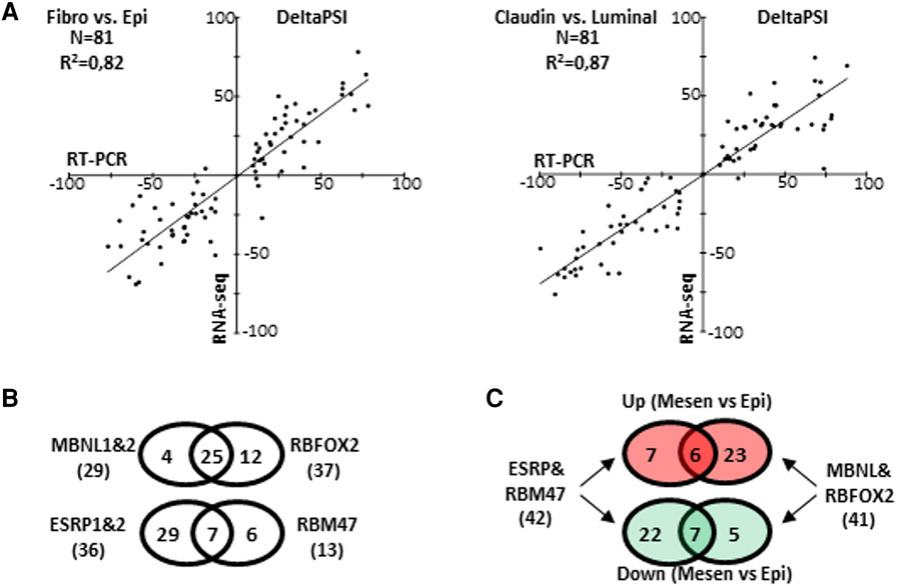
(*A*) Comparison of the percent splicing inclusion (psi) rate variations (deltaPSI) of the 81 exons of the “Mes-Epi” list, as measured by RT-PCR (*x*-axis) and by RNA-seq (*y*-axis). Exons are differentially spliced between normal fibroblast (Fibro) and normal epithelial (Epi) cells (*left* panel) and between Claudin-Low (mesenchymal-like) versus luminal (epithelial-like) breast cancer cells (*right* panel). (*B*) Venn diagram representing exons regulated by MBNL1&2 and RBFOX2 in MDA-MB-231 cells or regulated by ESRP1&2 and RBM47 in MCF-7 cells. Significant collusion was observed among mesenchymal and epithelial splicing factors, respectively. (*C*) Venn diagram representing exons more (red circles) or less (green circles) included in mesenchymal-like cells compared to epithelial-like cells and regulated by MBNL1&2, RBFOX2 (*right*), and ESRP1&2 and/or RBM47 (*left*).

To better predict the biological role of alternative splicing in epithelial and mesenchymal cells, we extracted from several large-scale data sets (Supplemental Table S1) a list of differentially spliced exons when comparing normal mesenchymal to normal epithelial cells and when comparing breast cancer mesenchymal-like cells (Claudin-low subtype) to breast cancer epithelial-like cells (luminal subtype). This established a list of 81 differentially spliced exons (Supplemental Table S4) that were initially validated by RT-PCR. A very good correlation was obtained when comparing RT-PCR and RNA-seq exon inclusion (percent spliced in [PSI]) rate variation (change in splicing/’delta PSI’) (Fig. 3A; Supplemental Fig. S1; Supplemental Table S4). In addition, using the same data sets, we identified six splicing factors whose expression differed when comparing epithelial- and mesenchymal-like cells. As already reported (Venables et al. 2013; Bebee et al. 2014; Mallinjoud et al. 2014; Vanharanta et al. 2014; Yang et al. 2016b), *ESRP1*, *ESRP2*, and *RBM47* genes were more expressed in epithelial-like cells as confirmed by RT-qPCR and Western blot analysis, while *MBNL1*, *MBNL2*, and *RBFOX2* genes were more expressed in mesenchymal-like cells (Supplemental Fig. S2, panels A to C). As shown on Figure 3B (Supplemental Figs. S2, S3; Supplemental Table S4), ESRP1 and ESRP2 depletion in epithelial-like cells switched the splicing pattern from an epithelial- to a mesenchymal-like pattern for 36 exons, as did RBM47 depletion for 13 exons. In contrast, MBNL1 and MBNL2 depletion in mesenchymal-like cells switched the splicing pattern from a mesenchymal- to an epithelial-like pattern for 29 exons, as did RBFOX2 depletion for 37 exons (Fig. 3B; Supplemental Figs. S2, S3; Supplemental Table S4). Some redundancy was observed since, for example, most exons regulated by MBNL1 and MBNL2 are also regulated by RBFOX2 (Fig. 3B). Most mesenchymal cell-enriched exons are regulated by MBNL1 and MBLN2 and/or RBFOX2, while most epithelial cell-enriched exons are regulated by ESRP1 and ESRP2 and/or RBFOX2 (Fig. 3C).

**Figure 4.**
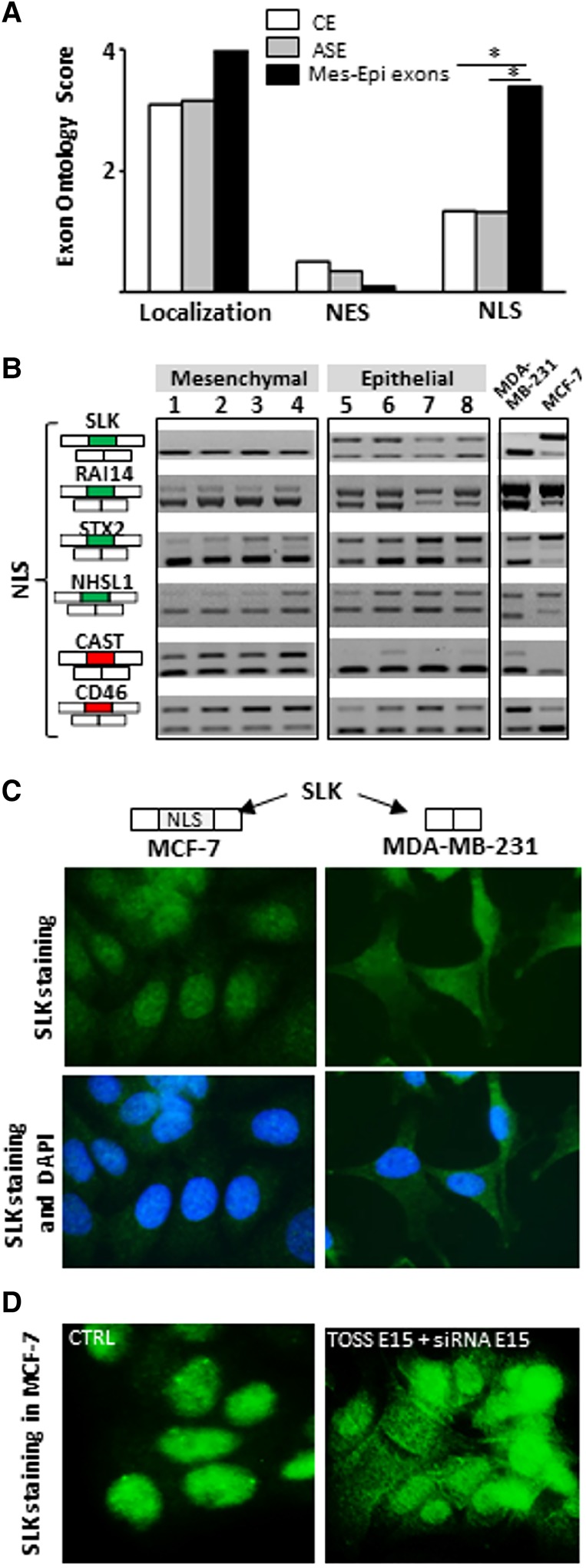
(*A*) Exons differentially spliced between epithelial-like and mesenchymal-like cells (Mes-Epi exons) code for protein segments that are enriched in “NLS” term when compared to constitutive (CE) or alternative (ASE) exons. (*) *P*-value < 0.05. (*B*) RT-PCR performed with total RNAs obtained from four normal epithelial (1 = HEPic, 2 = HPAEPic, 3 = HMEC, 4 = AG01134) and four normal mesenchymal (5 = HMF, 6 = HCFaa, 7 = AG0449, 8 = AG0450) cell lines and from MCF-7 and MDA-MB-231 breast cancer cell lines. The selected genes correspond to genes bearing alternative exons coding for protein segments containing nuclear localization signal (NLS). Red and green rectangles correspond to alternative exons with higher and lower inclusion rate, respectively, in mesenchymal-like cells compared to epithelial-like cells. (*C*) *SLK* exon 15 that encodes for a NLS is more often included in MCF-7 than in MDA-MB-231 cells (see panel *B*). Immunofluorescence of SLK protein indicates that SLK is more restricted to the nucleus in MCF-7 (epithelial-like) than in MDA-MB-231 (mesenchymal-like) cells. (*D*) Depletion of *SLK* transcripts that contain exon 15 (TOSS E15 + siRNA E15) leads to a more diffuse SLK staining within transfected MCF-7 cells compared to control (CTRL) cells.

Applying the Exon Ontology suite to the 81 selected exons (referred to below as the Mes-Epi exons list), we first noticed that all these exons are internal coding exons (“Mapping” in Supplemental Table S4) and encode for protein subcellular localization signals, protein–protein interacting domains, and/or phosphorylated peptides (“Exon annotations” in Supplemental Table S4). Interestingly, several protein features are selectively impacted by alternative splicing. For example, an enrichment for the “Nuclear Localization Signal” (NLS) term was observed (Fig. 4A; “Functional features” in Supplemental Table S4). This suggests that epithelial- and mesenchymal-like cells may express a similar set of proteins but with different subcellular localization (Fig. 4B). We sought to functionally validate this hypothesis, focusing on the Exon Ontology-identified putative NLS encoded by exon 15 of the *SLK* gene that produces a cytoplasmic kinase involved in cytoskeleton remodeling and cell migration (“Exon annotations” in Supplemental Table S4; Supplemental Fig. S4, panel A; Al-Zahrani et al. 2013). As *SLK* exon 15 is more often included in epithelial- than in mesenchymal-like cells (Fig. 4B, cf., for example, MDA-MB-231 to MCF-7 cells), we anticipated that SLK protein staining should be more pronounced in the nucleus of epithelial cells. As expected, immunofluorescence staining revealed a more restricted nuclear localization of SLK in MCF-7 (epithelial-like) than in MDA-MB-231 (mesenchymal-like) cells (Fig. 4C). To further challenge the role of *SLK* exon 15 coding sequence, MCF-7 cells were transfected with oligonucleotides inducing *SLK* exon 15 skipping (TOSS E15) combined with siRNA specifically targeting *SLK* exon 15 (siRNA E15), leading to the decrease of E15-containing transcripts (Supplemental Fig. S5, panel A). As predicted, the SLK protein staining in MCF-7 cells was less restricted to the nucleus in these conditions (Fig. 4D). Getting automated computational assistance for predicting protein features impacted by alternative splicing, as provided by Exon Ontology, will speed up the functional analysis of protein isoforms.

### Regulation of the AKT signaling pathway by epithelial cell-enriched splicing factors

**Figure 5.**
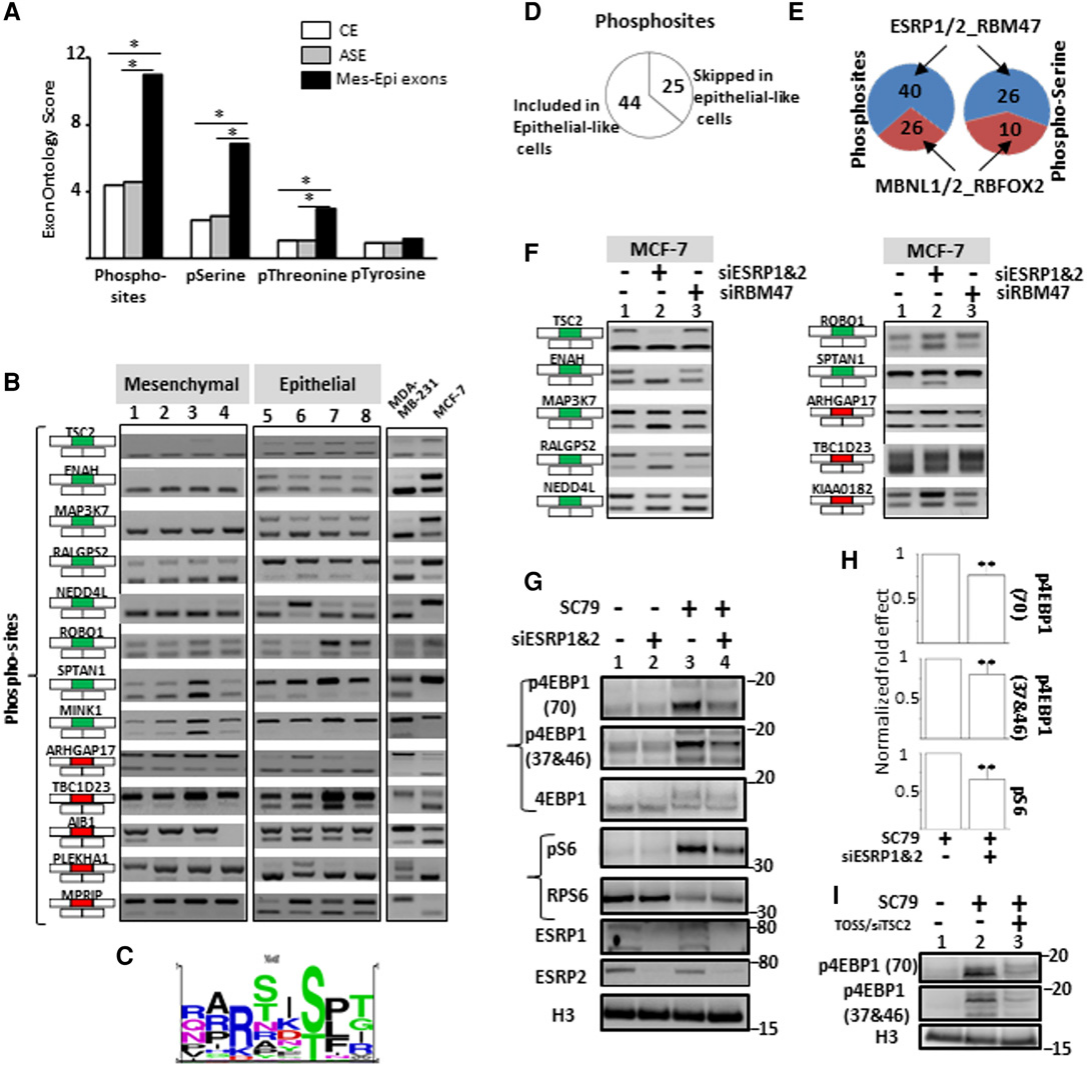
(*A*) Exons differentially spliced between epithelial- and mesenchymal-like cells (Mes-Epi exons) encode for protein segments that are enriched, when compared to constitutive (CE) or alternative (ASE) exons, for “Phosphorylated residue” (Phosphosites), “O-phospho-L-serine” (pSerine), “O-phospho-L-threonine” (pThreonine) terms but not for the “O4′-phospho-L-tyrosine” (pTyrosine) term. (*) FDR adjusted *P* value < 0.05. (*B*) RT-PCR performed with total RNAs obtained from four normal epithelial (1 = HEPic, 2 = HPAEPic, 3 = HMEC, 4 = AG01134) and four normal mesenchymal (5 = HMF, 6 = HCFaa, 7 = AG0449, 8 = AG0450) cell lines and from MCF-7 and MDA-MB-231 breast cancer cell lines. The selected genes correspond to genes bearing alternative exons coding for protein segments containing experimentally validated phosphosites. Red and green rectangles correspond to alternative exons with higher and lower inclusion rate, respectively, in mesenchymal-like cells compared to epithelial-like cells. (*C*) Sequence logo generated from the “PhosphoSite” website using sequences surrounding experimentally validated phosphorylated residues coded by exons differentially spliced between epithelial- and mesenchymal-like cells. (*D*) 44 and 25 experimentally-validated phosphorylated residues are encoded within exons more often included and less often included, respectively, in epithelial-like cells. (*E*) 40 and 26 experimentally validated phosphorylated residues are encoded within exons regulated by epithelial cell-enriched splicing factors (ESRP1, ESRP2, and RBM47) and by mesenchymal cell-enriched splicing factors (MBNL1, MBNL2, and RBFOX2), respectively (*left* panel). 26 and 10 experimentally validated phosphorylated serine residues are encoded within exons regulated by epithelial cell-enriched splicing factors (ESRP1, ESRP2, and RBM47) and by mesenchymal cell-enriched splicing factors (MBNL1, MBNL2, and RBFOX2), respectively (*right* panel). (*F*) RT-PCR performed with total RNAs extracted from epithelial-like MCF-7 cells transfected with control siRNAs (1), siRNAs targeting *ESRP1* and *ESRP2* (2) or *RBM47* (3). The selected genes correspond to genes bearing alternative exons encoding for experimentally validated phosphorylated residues. Red and green rectangles correspond to alternative exons with higher and lower inclusion rate, respectively, in mesenchymal-like cells compared to epithelial-like cells. (*G*) Western blot analyses of the phosphorylation pattern of proteins involved downstream of the AKT signaling pathway in epithelial-like MCF-7 cells transfected with control siRNAs or siRNAs targeting *ESRP1* and *ESRP2* and treated, or not, for 1 h with SC79 (AKT kinase activator). The p4EBP1(70) and p4EBP1(37&46) antibodies recognize phosphorylated residues on position 70, 37 and/or 46 of the E4BP1 protein. The pS6 antibody recognizes phosphorylated RPS6 protein. H3 (histone H3) is used as a loading control. (*H*) Quantification of Western blots shown in panel *G*. p4EBP1(70) and p4EBP1(37&46) signals were normalized by the signal obtained with an antibody recognizing both phosphorylated and unphosphorylated 4EBP1 protein (4EBP1). Likewise, the pS6 signal was normalized to total RPS6 signal (S6). (**) *P*-value < 0.005. (*I*) Western blot analyses of the phosphorylation pattern of 4EBP1 protein in MCF-7 cells transfected, or not, with TOSS and siRNAs targeting *TSC2* exon 27 (TOSS/siTSC2) and treated, or not, for 1 h with SC79. H3 (histone H3) is used as a loading control.

As already mentioned, the Exon Ontology database contains experimentally validated PTMs, including phosphorylation sites retrieved from several databases (see Methods). The Exon Ontology-based analysis revealed that the 81 selected exons are enriched for “Phosphorylated Residue,” “O-phospho-L-serine,” and “O-phospho-L-threonine” terms, but not for the “O4'-phospho-L-tyrosine” term when compared to CEs or ASEs (Fig. 5A; “Functional Features” in Supplemental Table S4). About one third (i.e., 28) of the protein segments coded by the 81 Mes-Epi exons contain at least one experimentally validated phosphorylation site (“PTM annotation” in Supplemental Table S4; Fig. 5B). Interestingly, the identified phosphosites are often associated with other protein features like subcellular localization or protein–protein interacting domains (Supplemental Fig. S4, panels B and C; Supplemental Fig. S5, panel B).

As the Exon Ontology web suite provides the sequences surrounding the PTM residues present in the selected exon set (“PTM annotation” in Supplemental Table S4), we looked for potential phosphorylation site consensus sequences in Mes-Epi exons using the PhosphoSite website (http://www.phosphosite.org/). Remarkably, the LOGO obtained is very similar to the AKT signaling pathway consensus sequence defined as RXRXXS/T (Fig. 5C; Toker 2008). We also noticed that a large proportion of the phosphorylation sites are encoded by exons that are more often included in epithelial-like cells (Fig. 5D) in an ESRP1/ESRP2-dependent manner (Fig. 5E,F).

Based on these observations, we tested whether the AKT signaling pathway is impacted by depletion of ESRP1 and ESRP2 in MCF-7 epithelial-like cells. Because the potential AKT-targeted phosphorylation sites frequently lie within exons that are skipped upon ESRP depletion (green exons on Fig. 5F), we anticipated that the AKT signaling pathway could be impaired in the absence of ESRP splicing factors. Indeed, ESRP1 and ESRP2 depletion specifically decreased the AKT-dependent phosphorylation of some of its targets, including 4EBP1 and RPS6 (also known as S6), after cell treatment with the SC79 AKT-activator (Fig. 5G, cf. lanes 4 and 3; Fig. 5H).

To test whether the ESRP-mediated effect on the AKT signaling pathway was a consequence of splicing regulation, we focused on the *TSC2* gene, which is known to play a major role in the AKT signaling pathway (Inoki et al. 2002; Cai et al. 2006; Toker 2008), and whose exon 27 is skipped upon ESRP-depletion (Fig. 5F). *TSC2* exon 27 skipping was induced in MCF-7 cells using oligonucleotides inducing *TSC2* exon 27 skipping combined with exon 27-specific siRNAs (Supplemental Fig. S5, panel A). Strikingly, this resulted in a decrease in AKT-mediated phosphorylation of 4EBP1, as did ESRP depletion (Fig. 5I, cf. lanes 3 and 2).

The Exon Ontology approach revealed that exons differentially spliced between epithelial- and mesenchymal-like cells code for protein segments containing phosphorylated residues (Fig. 5A,B) and that the splicing events regulated by ESRP1 and ESRP2 play an important role in the AKT signaling pathway in epithelial cells (Fig. 5C–F), as experimentally validated (Fig. 5G–I).

### Interplay between autophagy and mesenchymal cell-enriched splicing factors

**Figure 6.**
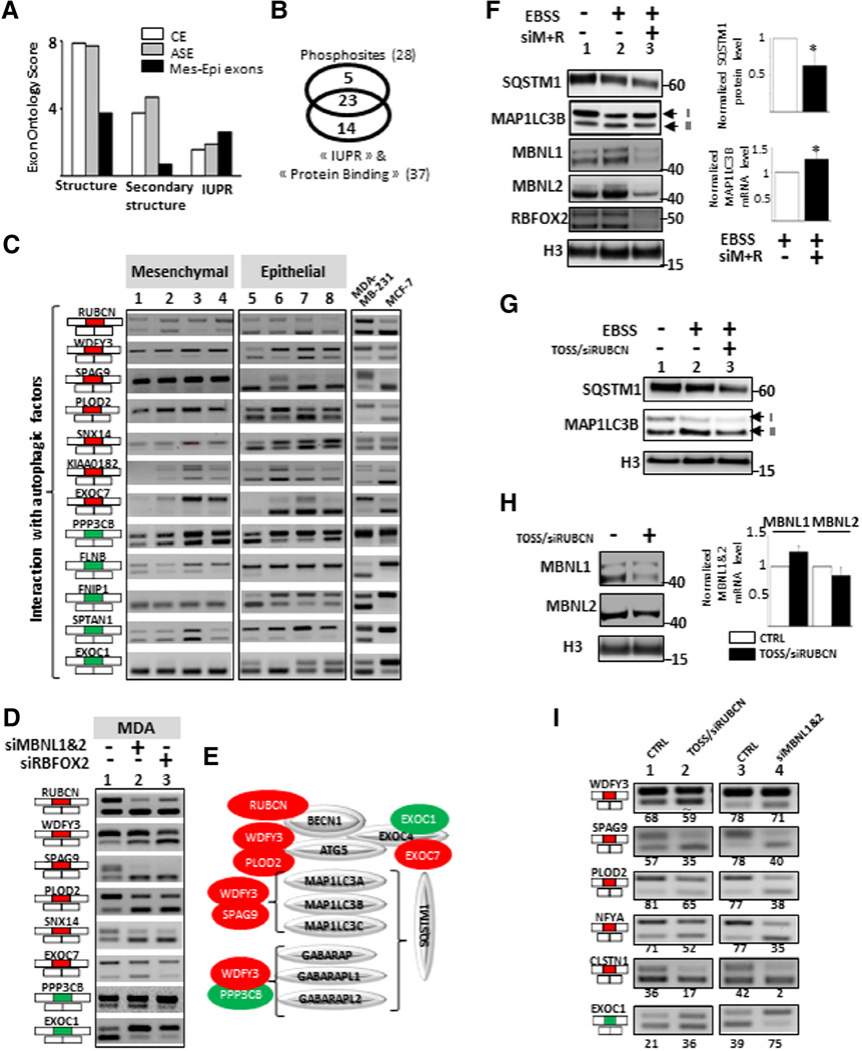
(*A*) Exons differentially spliced between epithelial- and mesenchymal-like cells (Mes-Epi exons) code for protein segments poorly associated with “structure” and “secondary structure” terms but that do contain unstructured regions (IUPR). (*B*) 28 and 37 exons out of the 81 selected exons code for protein segments containing experimentally validated phosphosites, IUPRs, and/or “Protein Binding” motifs, respectively; 23 of them contain both phosphosites and protein interacting motifs. (*C*) RT-PCR performed with total RNAs obtained from four normal epithelial (1 = HEPic, 2 = HPAEPic, 3 = HMEC, 4 = AG01134) and four normal mesenchymal (5 = HMF, 6 = HCFaa, 7 = AG0449, 8 = AG0450) cell lines and from MCF-7 and MDA-MB-231 breast cancer cell lines. The selected genes correspond to genes bearing alternative exons coding for protein interacting with autophagic factors. Red and green rectangles correspond to alternative exons with higher and lower inclusion rate, respectively, in mesenchymal-like cells compared to epithelial-like cells. (*D*) RT-PCR corresponding to genes with alternative exons and interacting with proteins involved in autophagy, using total RNAs obtained from mesenchymal-like MDA-MB-231 breast cancer cells transfected with control siRNAs (lane *1*), siRNAs targeting *MBNL1* and *MBNL2* (lane *2*), or *RBFOX2* (lane *3*). Red and green rectangles correspond to alternative exons with higher and lower inclusion rate, respectively, in mesenchymal-like cells compared to epithelial-like cells. (*E*) Genes with exons regulated by mesenchymal cell-enriched splicing factors produce proteins interacting with proteins involved in autophagy. Red and green proteins correspond to genes with alternative exons with higher and lower inclusion rate, respectively, in mesenchymal-like cells compared to epithelial-like cells. (*F*) Western blot analyses of SQSTM1, MAP1LC3B, MBNL1, MBNL2, and RBFOX2 in control (Earle's Balanced Salt Solution−[EBSS−]) or serum starved (EBSS +) MDA-MB-231 cells transfected with control siRNAs or siRNAs targeting *MBNL1*, *MBNL2*, and *RBFOX2* (siM+R). H3 (histone H3) is used as a loading control. The quantification of the SQSTM1 Western blot signal and *MAP1LC3B* mRNA level by RT-qPCR is shown on the right. (*) *P*-value < 0.05. (*G*) Western blot analyses of SQSTM1 and MAP1LC3B in control (EBSS−) or serum starved (EBSS +) MDA-MB-231 cells transfected with TOSS and siRNA targeting *RUBCN* exon 14 (TOSS/siRUBCN). H3 (histone H3) is used as a loading control. (*H*) MDA-MB-231 cells were transfected with TOSS and siRNA targeting *RUBCN* exon 14 (TOSS/siRUBCN). Western blot analysis of MBNL1 and MBNL2, with H3 (histone H3) used as a loading control (*left* panel). RT-qPCR analysis of the *MBNL1* and *MBNL2* mRNA levels in the same experimental conditions (*right* panel). (*I*) RT-PCR analysis using total RNAs extracted from mesenchymal-like MDA-MB-231 cells transfected as described in panel *H* or transfected with siRNAs targeting *MBNL1* and *MBNL2* (siMBNL1&2).

In analyzing the protein features encoded by the Mes-Epi exons, we noticed that the EXONT scores corresponding to “Structure” and “Secondary structure” terms were low compared to CEs or ASEs, while the IUPR score was slightly higher (Fig. 6A). This is interesting, as intrinsically disordered protein regions play an important role in protein–protein interactions that are regulated by phosphorylation (Fukuchi et al. 2011; Colak et al. 2013; Oldfield and Dunker 2014; Uversky 2015). Remarkably, more than 82% of the phosphorylation sites present in the 81 exons lie within IUPRs and/or annotated “protein binding” regions (Fig. 6B; Supplemental Table S4; Supplemental Fig. S4, panel C). In addition, these regions contain “P-rich” and “RXXK” motifs that are recognized by proteins like GRB2 containing SH3 domains (Supplemental Fig. S6, panel A; Supplemental Table S4; Belov and Mohammadi 2012). The co-occurrence of phospho-residues, IUPRs, and/or protein binding motifs in the protein segments coded by the 81 Mes-Epi exons suggested that alternative splicing of these exons may affect protein–protein interaction networks.

We therefore looked within the IntAct database (http://www.ebi.ac.uk/intact) for the partners of the 81 proteins harboring differentially spliced Mes-Epi exons. Interestingly, these partners are involved in biological processes relying on “nonmembrane-bounded organelles,” “vesicles,” “autophagy vacuole,” and “exocytosis” (Supplemental Fig. S6, panels B and C). In addition, several genes bearing exons regulated by mesenchymal cell-enriched splicing factors interact with autophagic factors (Fig. 6C–E). This includes the *RUBCN* gene (named *KIAA0226* in FasterDB and Exon Ontology) that codes for a major autophagy inhibitor interacting with beclin 1 (BECN1) and the *WDFY3* gene (also known as *ALFY*) that codes for an important adaptor protein for selective autophagy interacting with MAP1LC3B (also known as LC3B), SQSTM1 (also known as p62), and GABARAPs (Matsunaga et al. 2009; Isakson et al. 2013; Lamb et al. 2013; Baixauli et al. 2014; Wild et al. 2014; Khaminets et al. 2016; Ktistakis and Tooze 2016).

Based on these observations, we investigated the role of mesenchymal cell-enriched splicing factors in autophagy, a process involved in the degradation and recycling of cellular components, in particular, under cellular starvation (Matsunaga et al. 2009; Isakson et al. 2013; Lamb et al. 2013; Baixauli et al. 2014; Wild et al. 2014; Khaminets et al. 2016; Ktistakis and Tooze 2016). Autophagy is a dynamic process of intracellular bulk degradation in which cytosolic proteins and organelles are sequestered into double-membrane vesicles called autophagosomes, which are then fused with lysosomes for degradation and recycling. Autophagy receptors such as SQSTM1 recognize autophagic cargos and mediate formation of autophagosomes via binding to small ubiquitin-like modifiers such as MAP1LC3B and GABARAPs (Matsunaga et al. 2009; Isakson et al. 2013; Lamb et al. 2013; Baixauli et al. 2014; Wild et al. 2014; Khaminets et al. 2016; Ktistakis and Tooze 2016). The effect of depleting mesenchymal cell-enriched splicing factors on autophagy was tested by Western blot analysis of MAP1LC3B (whose level of lipidation can be traced by the appearance of the MAP1LC3B-II form) and of the autophagy receptor SQSTM1 which is a standard marker of cellular autophagy activity as it is degraded in the autophagosome with its cargos (Matsunaga et al. 2009; Isakson et al. 2013; Lamb et al. 2013; Baixauli et al. 2014; Wild et al. 2014; Khaminets et al. 2016; Ktistakis and Tooze 2016).

As shown on Figure 6F, depletion of MBNL1, MBNL2, and RBFOX2 (siM+R) in MDA-MB-231 cells affected both the SQSTM1 and MAP1LC3B protein expression patterns. In particular, serum starvation with Earle's Balanced Salt Solution (EBSS), which is classically used to activate autophagy, induced a decrease of SQSTM1, which was further decreased upon depletion of mesenchymal cell-enriched splicing factor (Fig. 6F, left panel, cf. lanes 3 and 2; see right panel for quantification). This effect was not due to a decrease in *SQSTM1* mRNA level (Supplemental Fig. S5, panel C). Depletion of mesenchymal cell-enriched splicing factors under starvation conditions also affected the MAP1LC3B protein expression pattern as it induced a slight increase and decrease of the levels of MAP1LC3B-I and MAP1LC3B-II forms, respectively, when compared to EBSS treatment alone (Fig. 6F, cf. lanes 3 and 2). This could result from MAP1LC3B-II degradation along with SQSTM1, since we observed an increase in total *MAP1LC3B* mRNA levels (Fig. 6F, right panel), which, in turn, may contribute to the slight MAP1LC3B-I form increase. Altogether, these results show that, in the absence of MBNL1, MBNL2 and RBFOX2, autophagy is stimulated, as evidenced by SQSTM1 and MAP1LC3B-II protein levels.

To test whether the effect of mesenchymal cell-enriched splicing factors was a consequence of splicing regulation, we focused on the *RUBCN* gene whose exon 14 is included in a MBNL1/2- and RBFOX2-dependent manner (Fig. 6D). Skipping of *RUBCN* exon 14 was forced in MDA-MB-231 cells using oligonucleotides inducing *RUBCN* exon 14 skipping and exon-specific siRNAs (Supplemental Fig. S5, panel A). Remarkably, *RUBCN* exon 14 skipping mimicked the effect of depletion of mesenchymal cell-enriched splicing factors, further reducing the SQSTM1 protein level under serum starvation (Fig. 6G, cf. lanes 3 and 2). A similar effect was observed by inducing the skipping of *WDFY3* exon 46 that is also regulated by mesenchymal cell-enriched splicing factors (Fig. 6D; Supplemental Fig. S5, panel D).

Interestingly, manipulation of *RUBCN* exon 14 splicing also resulted in the decrease of MBNL1 and MBNL2 protein levels (Fig. 6H, left panel), without affecting their mRNA level (Fig. 6H, right panel). Remarkably, it also mimicked the splicing effects induced by MBNL1/2 silencing (Fig. 6I). These results support a model where mesenchymal cell-enriched splicing factors control alternative splicing of autophagic regulators that, in turn, regulate MBNL1 and MBNL2 protein expression level.

Because a computational approach allowing prediction of the protein features affected by alternative splicing will be useful to the research community, we created a freely available web interface (http://fasterdb.ens-lyon.fr/ExonOntology/) that, after uploading the genomic coordinates of selected exons, gives access to the information stored in the Exon Ontology database, allows acquisition of potentially enriched protein features, and retrieval of relevant protein–protein networks (Supplemental Fig. S7).

## Discussion

Being able to routinely measure splicing variation at the RNA level, the main challenge is now to determine how these variants drive physiological and pathological cellular phenotypes. To address this challenging task, we methodically associated human exons to encoded protein features (named EXONT terms) using an ontology tree approach and already defined ontology terms (Montecchi-Palazzi et al. 2008; Mungall et al. 2011; Gene Ontology Consortium 2015; Mitchell et al. 2015). We also implemented an EXONT *Z*-score allowing measurement of a potential EXONT term enrichment within a list of selected exons compared to the appropriate set of control exons (Figs. 1, 2). Because we showed that different exon categories (e.g., first, internal, or last coding exons, constitutive and alternative exons) are differentially enriched for specific EXONT terms (Fig. 2), we want to stress here that it is important to compare a list of selected exons to the appropriate control list. For example, if one aims to identify EXONT terms enriched in a selected list of coregulated exons, it is advisable to compare exons corresponding to alternative promoters to the “First coding exons” category and to compare splicing regulated exons to either constitutive or alternative internal coding exons. The Exon Ontology web suite provides support for this specific step (Supplemental Fig. S7, panels C and H).

Applying Exon Ontology to a list of exons differentially spliced between epithelial- and mesenchymal-like cells, we uncovered specific protein features affected by alternative splicing (e.g., NLS) (Fig. 4; Supplemental Fig. S4, panel A). A dedicated table (“Exon annotations” in Supplemental Fig. S7, panel E) is automatically generated on the Exon Ontology website and describes all the protein features encoded by each of the analyzed exons. Of note, each protein annotation can be visualized on the FasterDB protein website (Supplemental Fig. S4). These resources will therefore speed up the characterization of biological consequences resulting from splicing variation.

This approach also revealed common protein features encoded by coregulated exons (e.g., phosphosites) (Fig. 5). In this setting, the Exon Ontology website generates a table containing the enrichment *Z*-scores for the most frequent protein features associated with the tested exons (“Functional features” in Supplemental Fig. S7, panels H and I). Finally, the Exon Ontology approach is useful to uncover protein features co-occurring within a set of coregulated alternative exons. For example, we observed that many phosphosites are embedded within protein–protein interaction domains (e.g., protein binding motifs, intrinsically disordered regions) (Fig. 6B). This observation suggests that Mes-Epi exons code for protein segments playing a role in the regulation of protein interaction networks. Integrating alternative splicing and interactome data sets allowed the identification of biological processes impacted by alternative splicing, as we uncovered an intricate relationship between autophagy and alternative splicing: splicing factors and alternative splicing events impact autophagy (Fig. 6F,G), and autophagic regulators impact splicing factor expression and splicing decisions (Fig. 6H,I). However, further experiments will be required to determine whether autophagy regulates directly or indirectly the MBNL1 and MBNL2 protein expression level. To provide users with useful information for investigating functional consequences of alternative splicing variation, the Exon Ontology web resource provides the list of proteins interacting with the products of the genes bearing tested alternative exons (“Protein–protein network” in Supplemental Fig. S7, panel G).

We noticed that some splicing-regulated genes share the same interacting partners (Fig. 6E; Supplemental Fig. S6, panel C) and that, in such a case, the regulated exons encode for similar protein sequences (Supplemental Fig. S6, panel D). For example, the *WDFY3* and *PLOD2* genes, whose products both interact with ATG5 (Fig. 6E), contain alternative exons that share strong sequence similarity (Supplemental Fig. S6, panel D). The same is true for alternative exons 11 and 7 of *EXOC1* and *EXOC7* genes, respectively, whose protein products interact with EXOC4 (Fig. 6E; Supplemental Fig. S6, panel D). These observations support a model where alternative exons play a role in regulating the competition in protein interaction since *EXOC1* exon 11 and *EXOC7* exon 7 are regulated in an opposite manner: *EXOC1* exon 11 is frequently included in epithelial cells and is repressed by mesenchymal splicing factors, while *EXOC7* exon 7 is more often included in mesenchymal cells and is positively regulated by mesenchymal splicing factors (Fig. 6D). Therefore, although further experiments are needed, comparing the protein sequences encoded by coregulated exons or exons that are inversely regulated could help to identify important functional amino acid residues.

Combined with the effort of the research community to characterize alternative splicing-dependent protein interaction networks (Corominas et al. 2014; Raj et al. 2014; Li et al. 2015; Tseng et al. 2015; Will and Helms 2016; Yang et al. 2016a) and with web services allowing the association of splicing events to protein feature annotation (Li et al. 2014; Rodriguez et al. 2015; Mall et al. 2016), the Exon Ontology website will be a useful tool for experimental biologists by providing computational support to help in the prediction of the biological consequences resulting from splicing variation.

## Methods

### Ontology tree

Ontological terms were selected from the Sequence Ontology–SO (version 1.45 25:08:2014), the Protein Modification Ontology–PSI-MOD (version 1.013.0 30:05:2014), and the InterPro tree and its GO mapping (interpro2go version 46.0). The original ontological trees were linked to eight main classes of protein features.

### Annotations

Annotations were derived from reference tools and databases, including InterProScan (version 5.3-46.0) (Jones et al. 2014), TMHMM (version 2.0c) (Krogh et al. 2001), IntAct (May 2015) (Orchard et al. 2014), UniProt (Oct. 2014) (UniProt Consortium 2015) , dbOGAP (Mar. 2014) (Wang et al. 2011), hUbiquitome (Mar. 2014) (Du et al. 2011), PhosphoSitePlus (Apr. 2014) (Hornbeck et al. 2012), dbPTM (May 2013) (Huang et al. 2016), PhosphoELM (May 2013) (Dinkel et al. 2011), ProteomeScout (Mar. 2014) (Matlock et al. 2015), and D2P2 (Dec. 2014) (Oates et al. 2013). We only used the “experimental” data sets of UniProt, dbOGAP, and dbPTM3. Localization motifs were identified using a custom Perl (version 5.10.1) script based on regular expressions. These annotations were mapped at the exon level using our splicing database FasterDB and stored in a MySQL database (version 14.14 distribution 5.1.73).

### EXONT score, *Z*-score, and FDR

For a given exon and a given feature, the EXONT score is computed by dividing the feature size (in nucleotides) by the exon size (in kilo-nucleotides). Only the coding part of the exon is considered. When the feature only partially overlaps the exon, only this overlapping region is considered. The *Z*-score is based on the comparison of an EXONT score of interest (for a selected set of sequences) with the distribution of 1000 EXONT scores obtained with sequence sets of approximately the same size that are randomly generated from a control sequence set (for instance, from all first coding exons). This is only done for the EXONT terms that are annotated with at least 4% of the human exons (91 EXONT terms) (see Supplemental Table S2). The EXONT score distributions were generated offline for sequence sets of varying sizes (from 100 nucleotides to 32 kilo-nucleotides). The EXONT scores are log-normally distributed, so the log of the EXONT scores are used to compute the *Z*-scores. The FDR is computed using the Benjamini and Hochberg strategy.

### Web interface

The web interface is written in PHP and Javascript. It also relies on a set of Perl (version 5.20.2) scripts to interact with the MySQL database (version 14.14 distribution 5.5.49). The web server is run by Apache (version 2.4.10) on a Debian machine (version 8.5).

### Cell culture, treatment, and transfection

Cell culture of standard MCF-7 and MDA-MB-231 cells as well as transient transfection assays were performed essentially as described previously (Dardenne et al. 2012; Samaan et al. 2014). Sequences of siRNAs and TOSS are provided in Supplemental Table S1. AKT activation experiments were performed as follows: 24 h after siRNA transfection, cells were first starved in serum-free medium (Earle's Balanced Salts with Sodium medium, Sigma E3024 and E2888) for 16 h and then reactivated in medium containing 5 µg/mL of SC79 (pan-AKT activator by phosphorylation; S7863, Selleckchem) for 1 h.

### RNA analysis

RNA extraction, RT-PCR, and RT-qPCR were described previously (Dardenne et al. 2012; Samaan et al. 2014). qPCR data were normalized with the *RNA18S5* gene as a control. Statistical analyses on means were performed using Student's *t*-tests (unilateral, paired, *P* < 0.05). Primer sequences for PCR and qPCR are provided in Supplemental Table S1.

### Western blot analysis

Total cell extracts were lysed in “NP40 buffer” (50 mM Tris-HCl , 400 mM NaCl, 5 mM EDTA , 1% IGEPAL , 0.2% SDS) complemented with protease and phosphatase inhibitors (11836145001 and 04906837001, Roche) and then incubated on ice for 30 min. Extracts were then sonicated for 10 min (Diagenode Bioruptor, 10 cycles, 30′′ on / 30′′ off). Protein concentrations from total cell extracts were determined using a Pierce BCA Protein Assay kit (Thermo Scientific). Total cell extracts were run on 4%–12% Bis-Tris gels (Invitrogen) and transferred on nitrocellulose membranes (iBlot Gel Transfer Stacks Nitrocellulose, Invitrogen). Membranes were washed in TBST (20 mM Tris, pH 7.6 , 130 mM NaCl , 0.1% Tween 20) and blocked in 5% (w/v) dry nonfat milk or 5% (w/v) bovine serum albumin (Sigma) for primary phospho-antibodies. Membranes were then incubated with primary antibodies (overnight, 4°C) and washed before being incubated with secondary HRP-conjugated antibodies for 1 h. Primary and secondary antibodies are listed in Supplemental Table S1. Image acquisitions were performed using the ChemiDoc Touch Imaging System (Bio-Rad), and quantification was performed using Image Lab software (v.5.2.1, Biorad) and normalized with histone H3 or total nonphosphoprotein. Statistical analyses on means were made using Student's *t*-tests (unilateral, paired, *P* < 0.05).

### Immunofluorescence

Cells were fixed in 4% paraformaldehyde for 20 min. After three washes in 1× PBS, cells were permeabilized in 0.2% Triton X-100 for 30 min and left for 1 h in blocking solution (1× PBS, 15% serum, 0.1% Triton X-100). Slides were then incubated in blocking solution containing rabbit anti-SLK (1/100; ab65113, Abcam) primary antibody (overnight, 4°C). After three washes in 1× PBS, slides were incubated 2 h in blocking solution containing FITC-conjugated anti-rabbit IgG (1/2000; Sigma), and nuclei were stained with DAPI (10 nM final, 10 min).

### Software availability

The Exon Ontology version used and released in this manuscript is v1.5.0. The ontology (i.e., the EXONT terms) and the human annotation (i.e., the association between EXONT terms and human coding exons) are available on the Exon Ontology website (except for the dbPTM data set, which is still available from the dbPTM website). In addition, the scripts to run Exon Ontology analyses from the command line are available in our GitLab repository (https://gitlab.com/ExonOntology/ExonOntology), and in Supplemental Data S1.

## Supplementary Material

Supplemental Material
