# Supplementary material for: Identification of protein features encoded by alternative exons using Exon Ontology
Source: Genome Res. 2017 Jun;27(6):1087–97. doi: 10.1101/gr.212696.116 (PMC5453322; doi:10.1101/gr.212696.116)
Supplement: Supplemental Material [file supp_27_6_1087__index.html]

Identification of protein features encoded by alternative exons using Exon Ontology — Supplemental Material 

# Identification of protein features encoded by alternative exons using Exon Ontology

## Supplemental Material

- Supplemental\_Fig\_S1.pdf
- Supplemental\_Fig\_S2.pdf
- Supplemental\_Fig\_S3.pdf
- Supplemental\_Fig\_S4.pdf
- Supplemental\_Fig\_S5.pdf
- Supplemental\_Fig\_S6.pdf
- Supplemental\_Table\_S1.xlsx
- Supplemental\_Table\_S2.xlsx
- Supplemental\_Table\_S3.xlsx
- Supplemental\_Table\_S4.xlsx
- Supplemental\_Data\_S1.zip
