# Supplementary material for: Identification of protein features encoded by alternative exons using Exon Ontology
Source: Genome Res. 2017 Jun;27(6):1087–97. doi: 10.1101/gr.212696.116 (PMC5453322; doi:10.1101/gr.212696.116)

## Exon Ontology: Functional Genomics At Exon Level Resolution

## Supplemental Figure S1

RT-PCR analysis of the 81 selected exons that are differentially spliced when comparing mesenchymal- and epithelial-like cells, using total RNAs from 4 normal mesenchymal cell types (Fibro), 4 normal epithelial cell types (Epi), 4 breast cancer mesenchymal-like cell types (Claudin low), and 4 breast cancer epithelial-like cell types (Luminal).

Supplemental Figure S1

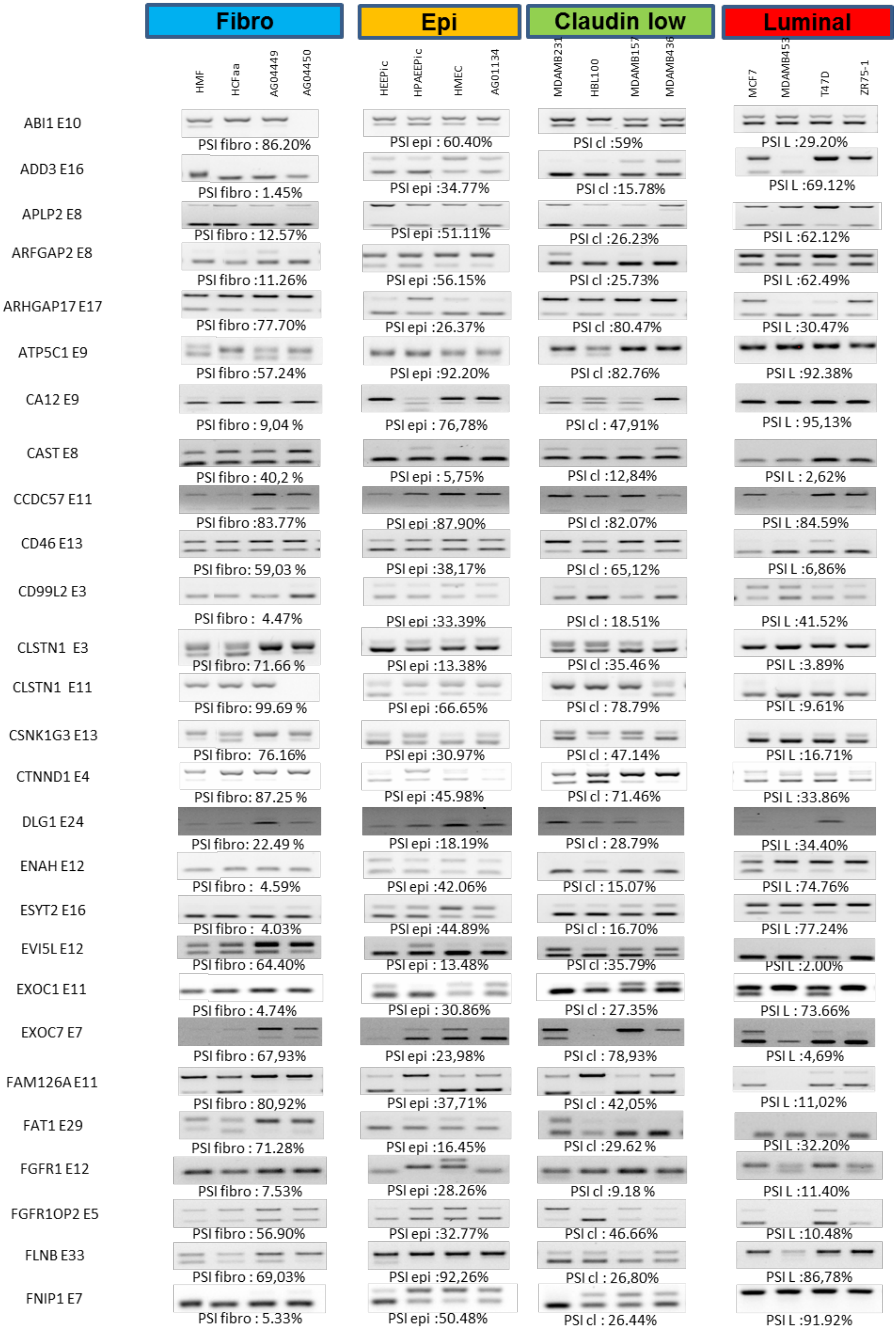

Supplemental Figure S1 (continued)

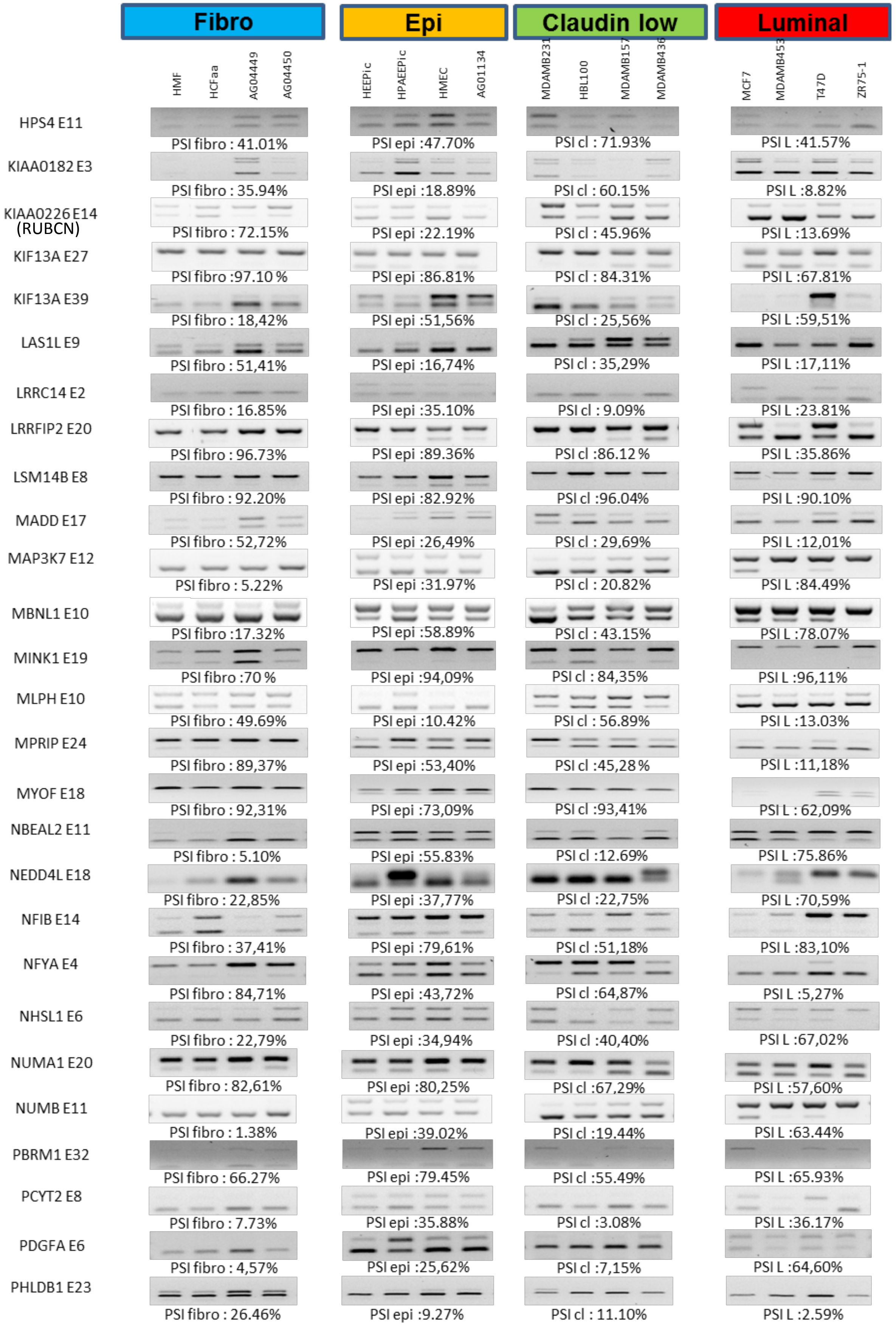

Supplemental Figure S1 (end)

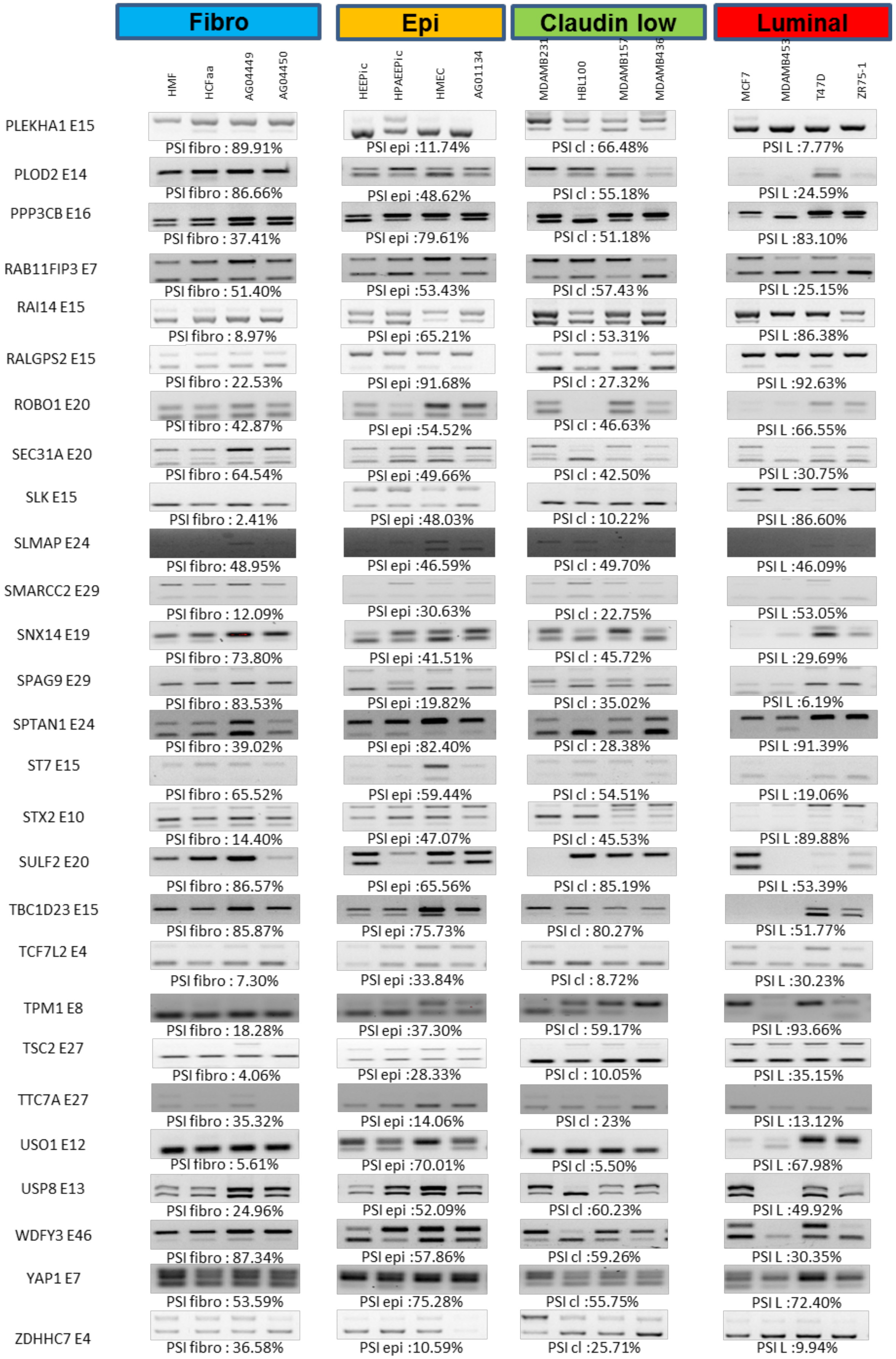

Supplement: Supplemental Material [file supp_gr.212696.116_Supplemental_Fig_S1.pdf]
