# Supplementary material for: Identification of protein features encoded by alternative exons using Exon Ontology
Source: Genome Res. 2017 Jun;27(6):1087–97. doi: 10.1101/gr.212696.116 (PMC5453322; doi:10.1101/gr.212696.116)
Supplement: Supplemental Material [file supp_gr.212696.116_Supplemental_Fig_S2.pdf]

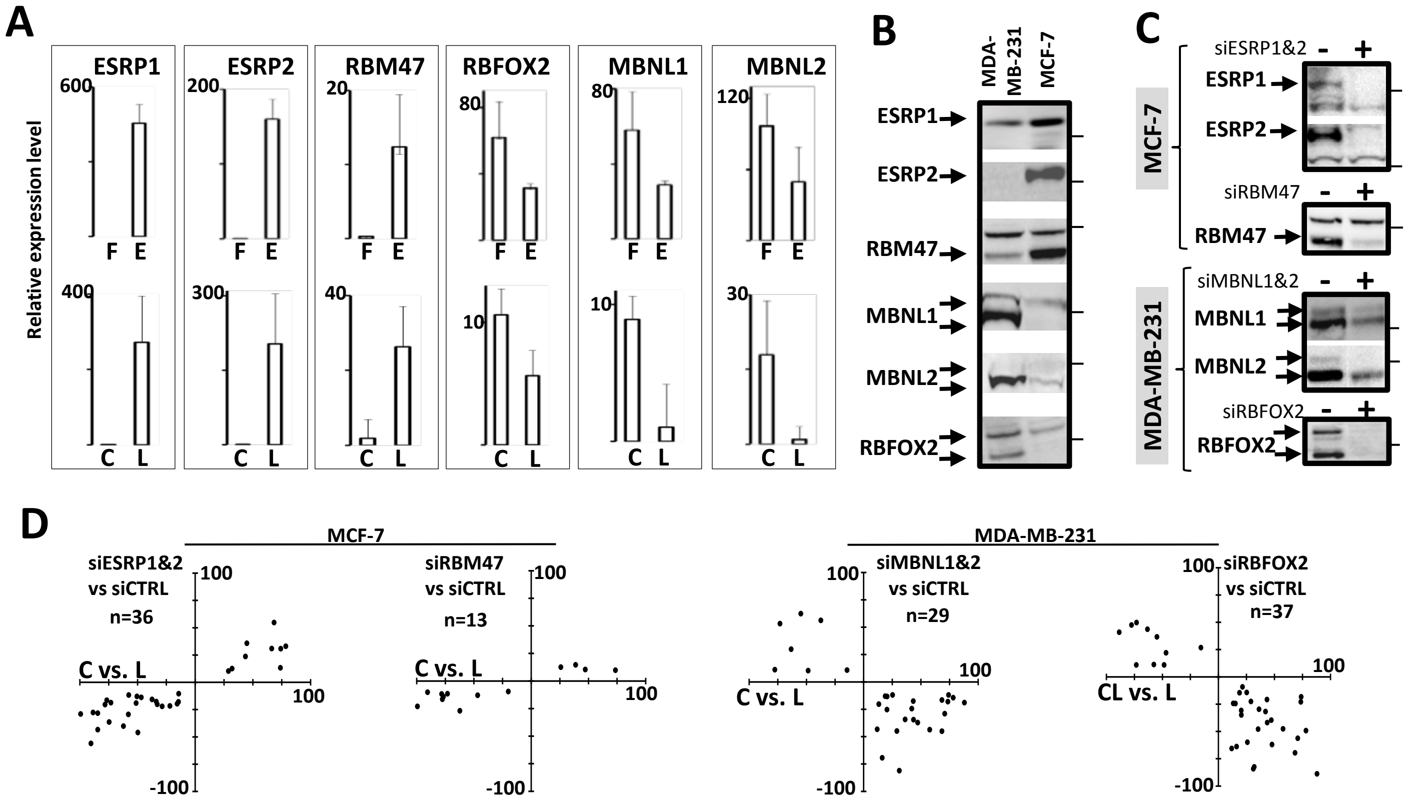

Supplemental Figure S2

A. Relative expression level (y-axis) of splicing factors measured by RT-qPCR in 4 normal fibroblast cells (F), 4 normal epithelial cells (E), 4 cancer claudin-low mesenchymal-like cells (C), or 4 cancer Luminal epithelial-like cells (L).

B. Western Blot analysis of splicing factor expression in MDA-MB-231 (mesenchymal-like cells) and MCF-7 (epithelial-like cells) breast cancer cells.

C. Western Blot analysis of splicing factor expression in MCF-7 breast cancer cells (epithelial-like cells) transfected with siRNAs targeting ESRP1 and ESRP2 (siESRP1&2) or RBM47 (siRBM47) and in MDA-MB-231 breast cancer cells (mesenchymal-like cells) transfected with siRNAs targeting MBNL1 and MBNL2 (siMBNL1&2) or RBFOX2 (siRBFOX2).

D. Comparison of the differential inclusion rate (deltaPSI, %) measured by RT-PCR after depletion of ESRP1 and ESPR2 (siESRP1&2), or RBM47 (siRBM47) in MCF-7 epithelial-like cells (y-axis, left panels) or after depletion of MBNL1 and MBNL2 (siMBNL1&2), or RBFOX2 (siRBFOX2) in MDA-MB-231 mesenchymal-like cells (y-axis, right panels) and when comparing mesenchymal-like to epithelial-like cells (C vs L, x-axis).
