# Supplementary material for: Identification of protein features encoded by alternative exons using Exon Ontology
Source: Genome Res. 2017 Jun;27(6):1087–97. doi: 10.1101/gr.212696.116 (PMC5453322; doi:10.1101/gr.212696.116)

Exon Ontology: Functional Genomics At Exon Level Resolution

Supplemental Figure S3

RT-PCR analysis of the 81 selected exons differentially spliced when comparing mesenchymal- and epithelial-like cells using total RNAs obtained from epithelial-like MCF-7 cancer cells transfected with siRNAs targeting ESRP1 and ESRP2 (siESRP1/2) or RBM47 (siRBM47) or from mesenchymal-like MDA-MB-231 breast cancer cells transfected with control siRNAs targeting MBNL1 and MBNL2 (siMBNL1/2) or RBFOX2 (siRBFOX2).

Supplemental Figure S3

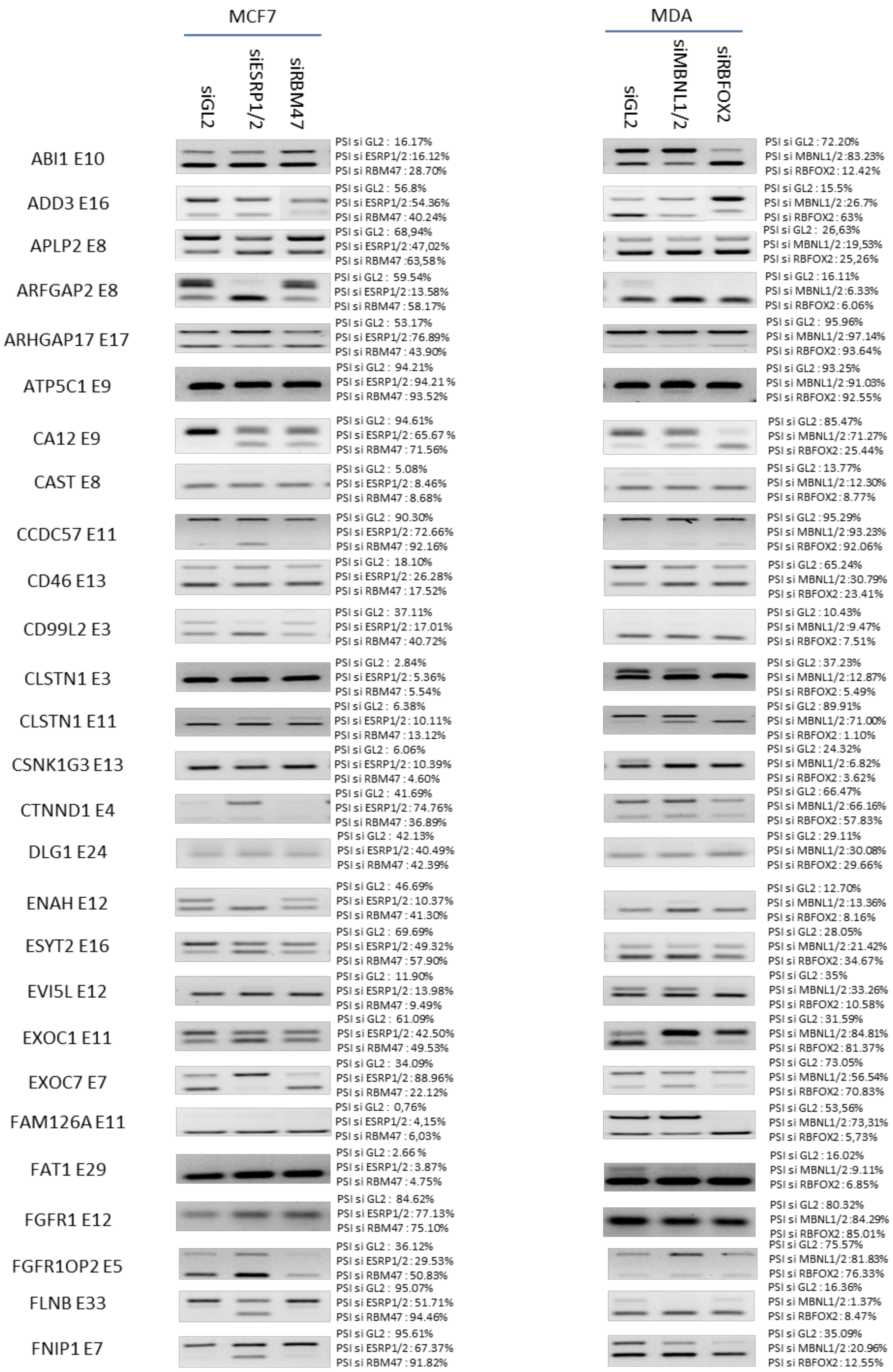

Supplemental Figure S3 (continued)

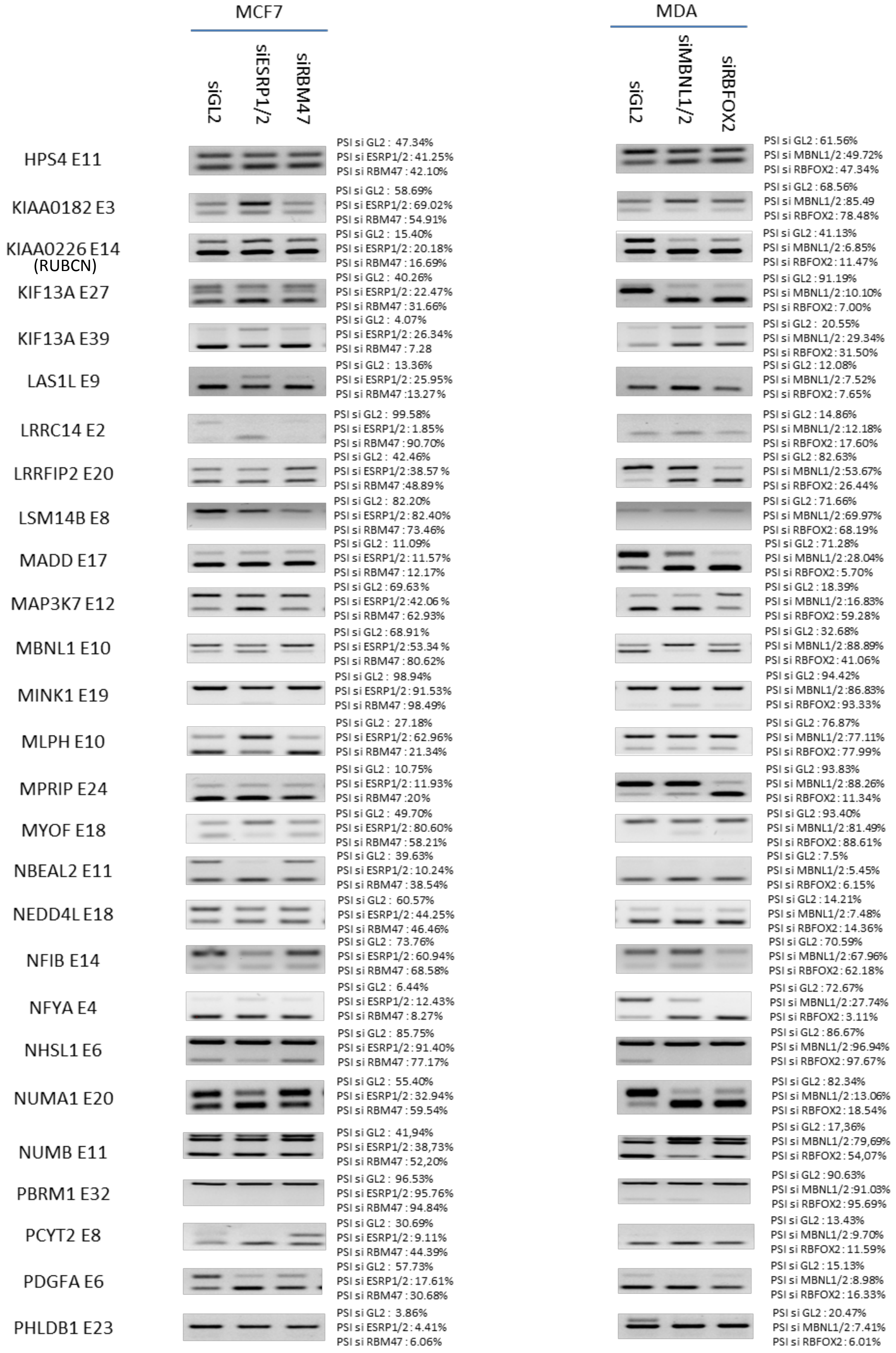

Supplemental Figure S3 (end)

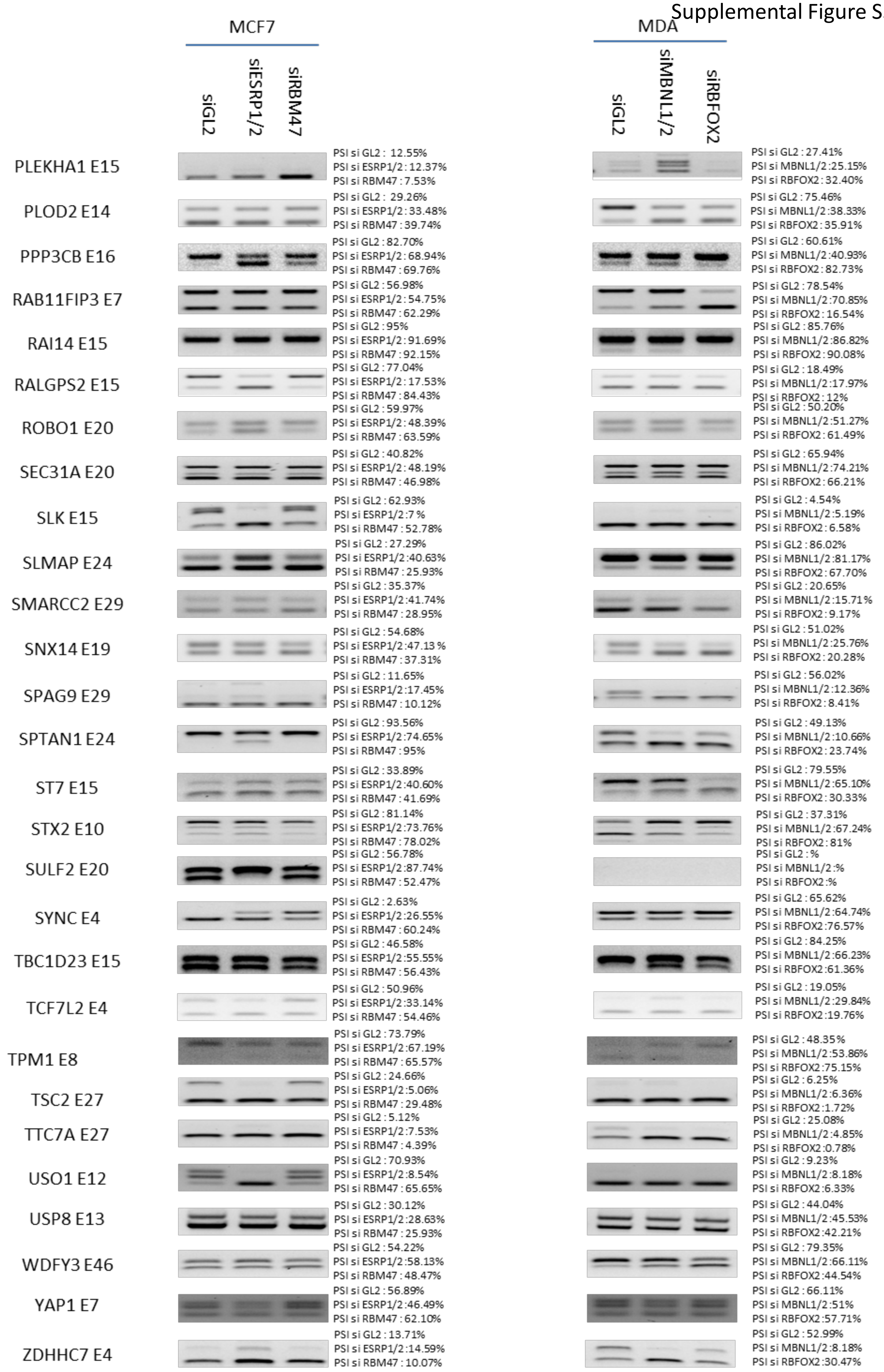

Supplement: Supplemental Material [file supp_gr.212696.116_Supplemental_Fig_S3.pdf]
