# Supplementary material for: Identification of protein features encoded by alternative exons using Exon Ontology
Source: Genome Res. 2017 Jun;27(6):1087–97. doi: 10.1101/gr.212696.116 (PMC5453322; doi:10.1101/gr.212696.116)

Exon Ontology: Functional Genomics At Exon Level Resolution

Supplemental Figure S4

A. “Faster DB protein” screenshot corresponding to the *SLK* gene illustrating the prediction of a Nuclear Localization Signal in exon 14.
B. “Faster DB protein” screenshot corresponding to the *RAI14* gene illustrating the prediction of a Nuclear Localization Signal in exon 15 co-occurring with experimentally validated phospho-serine and phospho-threonine sites.
C. “Faster DB protein” screenshot corresponding to the *TSC2* gene illustrating the prediction of an Intrinsically Unstructured Polypeptide Region in exon 27 co-occurring with experimentally validated phosphoserine sites.

Supplemental Figure S4

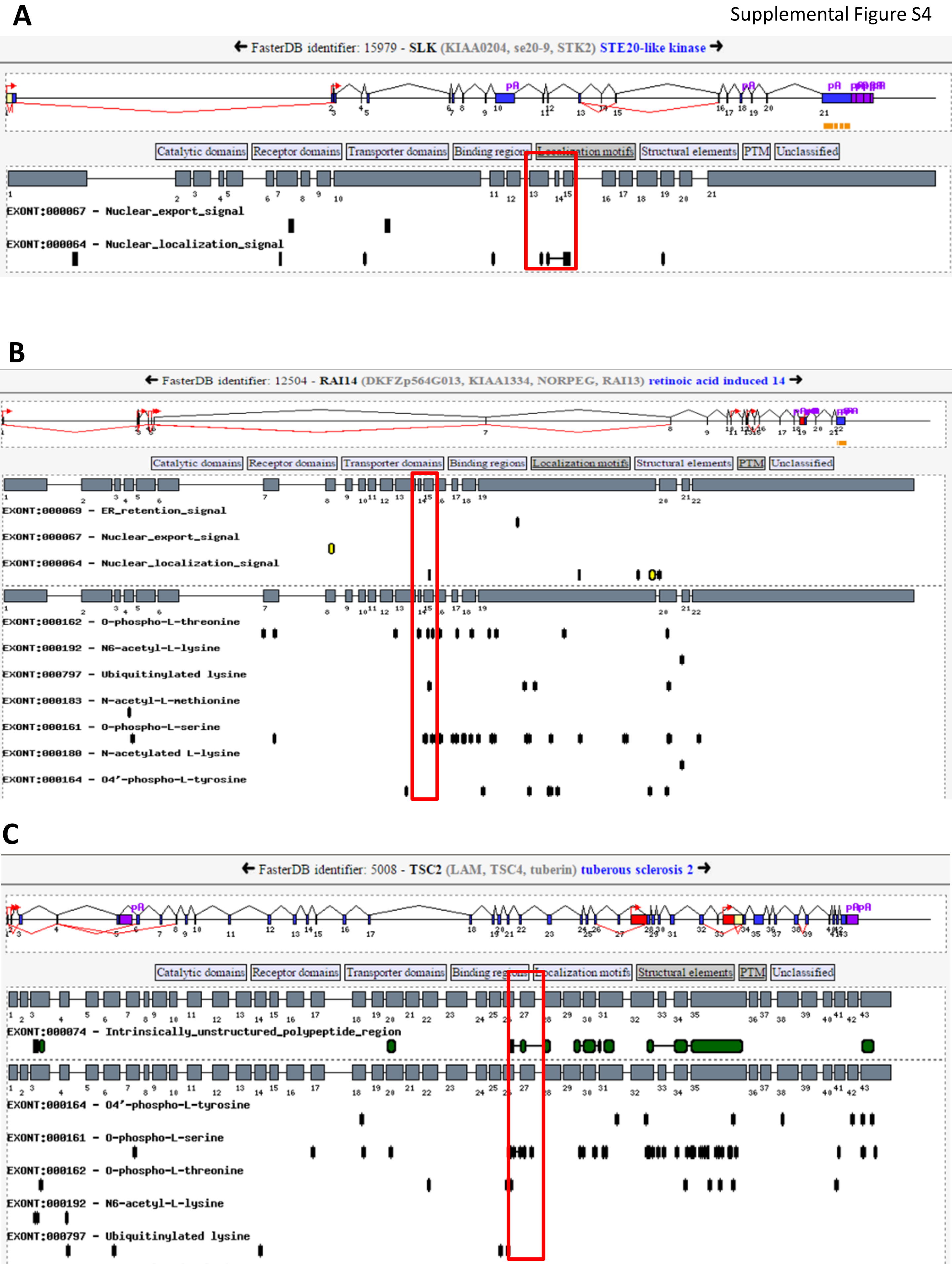

Supplement: Supplemental Material [file supp_gr.212696.116_Supplemental_Fig_S4.pdf]
