# Supplementary material for: Identification of protein features encoded by alternative exons using Exon Ontology
Source: Genome Res. 2017 Jun;27(6):1087–97. doi: 10.1101/gr.212696.116 (PMC5453322; doi:10.1101/gr.212696.116)
Supplement: Supplemental Material [file supp_gr.212696.116_Supplemental_Fig_S5.pdf]

Exon Ontology: Functional Genomics At Exon Level Resolution

Supplemental Figure S5

A. MCF-7 cells were transfected with an oligonucleotide inducing *SLK* exon 15 skipping (TOSS E15) and an siRNA targeting *SLK* exon 15 (siRNA E15) leading to the decreased level of exon 15-containing *SLK* transcript, or with oligonucleotides inducing *TSC2* exon 27 skipping (TOSS E27) combined with siRNAs targeting *TSC2* exon 27 (siRNA E27) leading to the decreased level of exon 27-containing *TSC2* transcript. MDA-MB-231 cells were transfected with oligonucleotides inducing *WDFY3* exon 46 skipping (TOSS E46) and siRNAs targeting WDFY3 exon 46 (siRNA E46) leading to the decreased level of exon 46-containing *WDFY3* transcript or with oligonucleotides inducing *RUBCN* exon 14 skipping (TOSS E14) and siRNAs targeting *RUBCN* exon 14 (siRNA E14) leading to the decreased level of exon 14-containing *RUBCN* transcript.
B. 28 and 30 exons of the 81 selected exons code for protein segments containing experimentally validated phospho-sites and/or protein sub-cellular localization signals ("Localization").
C. RT-qPCR analysis of SQSTM1 mRNA level of MDA-MB-231 cells transfected with a control siRNA (lanes 1 and 2) or siRNAs targeting *MBNL1* and *MBNL2* (lane 3) and incubated in normal growth medium (lane 1) or in the absence of serum (lanes 2 and 3).
D. Western blot analysis of SQSTM1 in control (EBSS-) or serum starved (EBSS +) MDA-MB-231 cell line transfected with TOSS and siRNA targeting *WDFY3* exon 46 (TOSS/siWDFY3). H3 (histone H3) is used as a loading control.
E. DAVID gene ontology analysis (https://david.ncifcrf.gov/) was performed using the genes bearing the 81 exons that are differentially spliced between epithelial and mesenchymal cells. Only major "Functional Annotation Clustering" are shown.

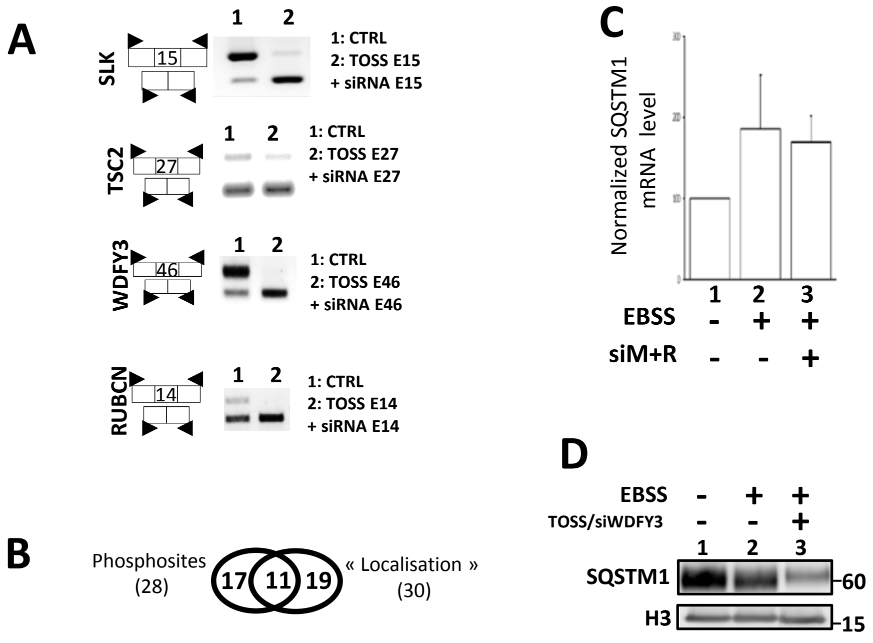

E

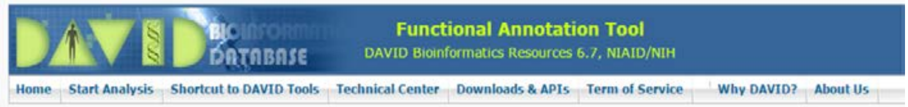

	Count	P_Value	Benjamini
GTPase regulator activity	10	6.9E-5	1.2E-2
nucleoside-triphosphatase regulator activity	10	8.2E-5	4.9E-3
cytoskeletal protein binding	11	6.9E-5	6.2E-3
cytoskeleton	16	6.6E-4	4.1E-2
protein localization	14	7.1E-5	5.6E-2
protein transport	13	7.6E-5	3.0E-2
