# Supplementary material for: Identification of protein features encoded by alternative exons using Exon Ontology
Source: Genome Res. 2017 Jun;27(6):1087–97. doi: 10.1101/gr.212696.116 (PMC5453322; doi:10.1101/gr.212696.116)

Exon Ontology: Functional Genomics At Exon Level Resolution

Supplemental Figure S6

A. Several genes differentially spliced between mesenchymal and epithelial cells produce proteins interacting with GRB2 and/or 14.3.3 proteins. pS/T means exons encoding phosphorylated peptides; “P-rich” means exons encoding peptides with proline-rich regions. The GRB2 protein interacts with P-rich regions or phospho-tyrosines (pY). The 14.3.3 proteins interact with phospho-serine or –threonine (pS/T). Red and green proteins correspond to genes with alternative exons more and less included, respectively in mesenchymal- than epithelial-like cells.
B. Proteins coded by genes containing the alternative exons that are differentially spliced when comparing mesenchymal- and epithelial-like cells interact with proteins involved in biological processes relying on “non-membrane bound organelle”, “vesicle”, “autophagic vacuole” and “exocytosis”. The analysis of GO term enrichment was performed with “DAVID Ontology” (https://david.ncifcrf.gov/).
C. Autophagy process derived from KEGG pathway (yellow proteins are autophagic factors) where genes with alternative exons differentially spliced when comparing mesenchymal- and epithelial-like cells are represented by a color code: red and green proteins correspond to genes with alternative exons more and less included, respectively in mesenchymal- than epithelial-like cells.
D. Protein sequence comparison between sequences encoded by alternative exons from genes involved in autophagy and sharing common partners. Red and green rectangles correspond to alternative exons that are more or less included, respectively, in mesenchymal- compared to epithelial-like cells.

Supplemental Figure S6

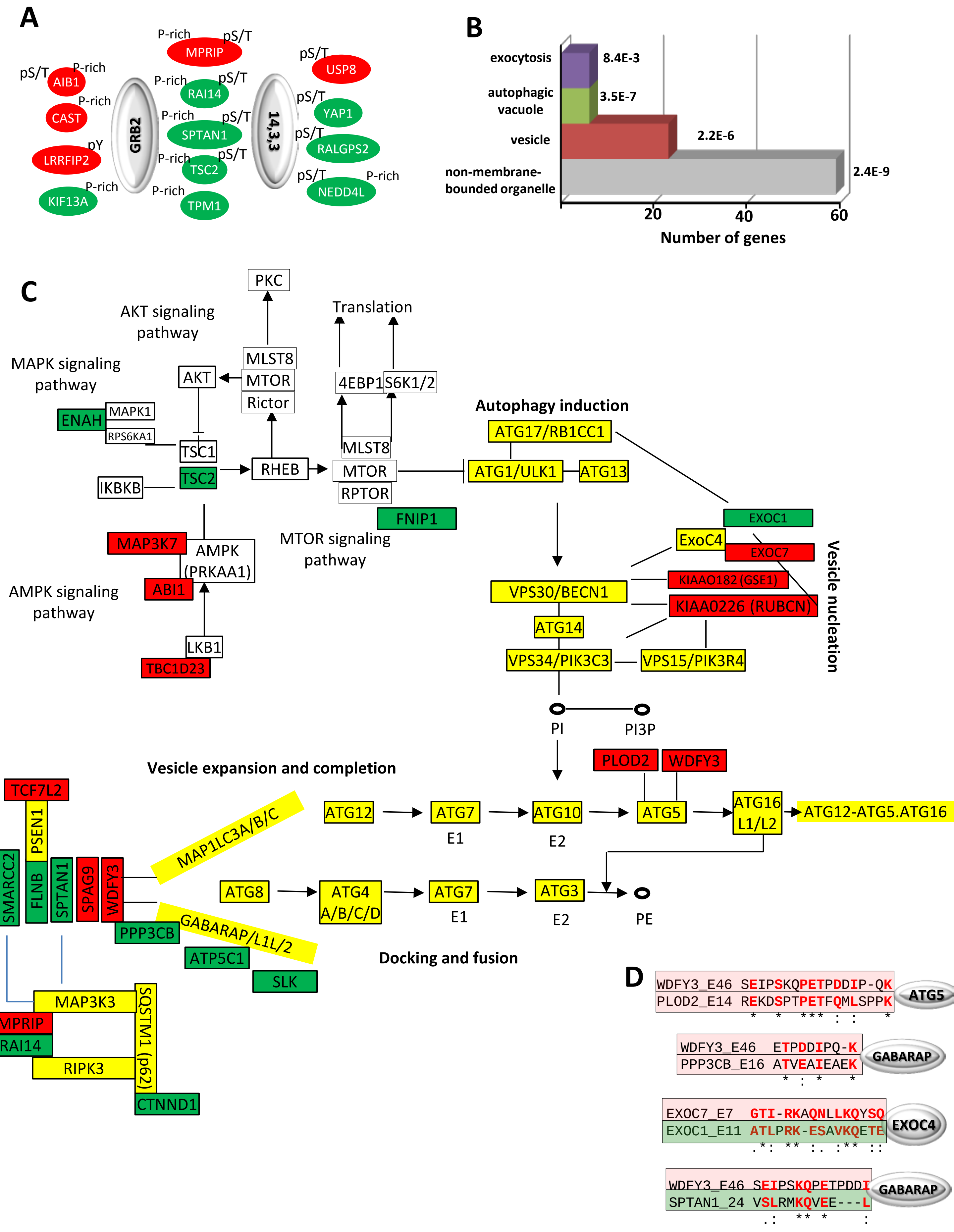

Supplement: Supplemental Material [file supp_gr.212696.116_Supplemental_Fig_S6.pdf]
